# Spirit of the English Periodicals, and Notices of English Medical Literature

**Published:** 1835-04-01

**Authors:** 


### Periscope;

OR,

### CIRCUMSPECTIVE REVIEW.

"Ore trahit quodcunque potest, atque addit acervo."

### I.

### Spirit of the English Periodicals, and Notices of English Medical Literature.

### Extraordinary Case of Tetano-Epileptic Convulsions.

A case of this kind lately occurred in practice, which exceeded in melancholy interest any thing ever witnessed by ourselves, during a very long course of observation and experience—and we believe we can say the same on the part of several other physicians and surgeons who were in professional attendance—among whom were Sir A. Cooper, Dr. Bright, Dr. Farre, Dr. Watson, Mr. Macintyre, Mr. Chisholme, and others.

The patient was a gentleman of large fortune, aged about 52 years, though apparently much older; but who had been remarkably active and strong till within the two or three last years of his life. At that time he fell from his horse, while at some distance from his mansion in Wales, in a kind of fit, the exact nature of which could not be ascertained, as it was over before medical attendance could be procured. It was considered to be epileptic, because he had subsequently several attacks of the epileptiform character. In the latter part of November last, Dr. Johnson was called into attendance on this gentleman, in consequence of a bronchitic affection under which he was labouring, and a very obstinate constipation of the bowels. The bronchitis was not very acute, and was readily relieved by the usual means. The bowels were also brought into a more manageable state by proper aperients, and the patient was so well as to go out in his carriage, and Dr. J. only called occasionally to see him. It was during this period that Dr. J. learnt some particulars of the epileptic seizures which had previously occurred. The patient, though not a man of much intellectual power, did not appear to have had his sensorial energies in the least degree impaired by the epileptic paroxysms.

On Friday night (26th Dec.) the patient, after some anxiety and want of sleep, occasioned by a domestic event, not of a mournful nature, was seized with a violent fit, or rather series of fits, which lasted several hours. They were evidently of the epileptic kind—and were followed by a semi-apoplectic sopor, such as often succeeds the epileptic convulsion. When the stupor subsided, the patient was perfectly sensible, and merely complained of soreness in the muscles, but no head-ache, except when he coughed. His bowels were opened by medicine, and it was hoped that the paroxysm would not return. In this hope we were grievously disappointed. It was only the first of a series of paroxysms that attacked this unfortunate gentleman, with more or less violence, and with longer or shorter intervals, from the 26th December till the second day of January, when death put an end to the most dreadful sufferings which patient ever endured, or physician witnessed in this world!

Although the paroxysms varied considerably, in respect to intensity, duration, and intervals; yet they were so identical in kind, that the description of one may serve for all. The premonition was a quivering sensation in the muscles of the lower extremities, which caused the patient to cry out—"the spasms are coming on—hold me, hold me!" He then groaned loudly, till the spasms took away all power of speech. The face was drawn violently towards the *left* side, as were the eyes. The left arm was bent, and drawn across the chest, the fist being clenched. The left leg and thigh were drawn upwards, and bent across the other leg and thigh. Thus the flexors and adductors of the left extremities overcame the extensors and abductors; but not by one uniform convulsive contraction, as in tetanus, but by a series of the most rapid and painful vibrations. These vibrations could not be counted; but they were perfectly cognizable by the eye and the touch. The muscles of the other side of the body, and of the limbs, underwent similar convulsive vibrations, but were not rigidly contracted at the same time, as on the left side. Thus the abdominal muscles could be seen and felt in this convulsive and vibratory state, and it was evident, from the respiration and the voice (when the latter was audible), that the diaphragm suffered in common with the muscles of voluntary motion. There never was well-marked opisthotonos or embrosthotonos, though the muscles on the front of the body were more convulsed and contracted than those on the back, with the exception of the large muscles about the back of the neck, which generally drew the head backwards, while the face and eyes were drawn to the left. After all the severe paroxysms, the left arm remained, for a time, completely paralytic. If the interval was of some duration, it regained sense and motion in a considerable degree.

The paroxysms were very various in duration—lasting from one minute to fifteen or twenty. They differed much in intensity; since the patient, in some attacks, retained his consciousness, and in others he was totally insensible, and remained so for many minutes after the paroxysm was over. Whenever the consciousness did return, whether immediately on the cessation of the spasms, or after a short lapse of time, the intellectual functions were as clear as at any period of the patient's life. The intervals were also very various as to duration. Sometimes they extended to several hours—at other times, they actually ran into one another—one paroxysm having scarcely ceased, when another commenced. In every attack, the pulse rapidly increased in frequency, and became smaller in calibre. In the severe paroxysms, it was almost imperceptible, and quite innumerable. After the paroxysm, it gradually became developed, full and strong.

In all the severe paroxysms, the breathing became dreadfully laborious, quick, and wheezing, Many times we expected that the patient would expire, from the collection of mucus in the trachea and bronchia, and from the inadequate supply of air to the lungs. These attacks continued day and night, with longer and shorter intervals, till the 29th of December, when, after a series of frightful paroxysms, the patient fell, apparently, into the "agonies of death," or rather into that state of insensibility which precedes dissolution. The breathing was laborious and stertorous—dead rattles in the throat—eyes open and inanimate—total paralysis of the whole body—mouth wide open—evacuations passed involuntarily—skin cold and clammy—in short, he appeared *in articulo mortis*, from which not one of his medical attendants conceived a possibility of emergence. Several of them waited, hour after hour, to see the final close. This state lasted six hours and upwards. In the course of this period, we occasionally perceived a slight tremor or quivering in the tendons of the wrist, during which, as in the severe paroxysms, the pulse became nearly extinct, the breathing short, broken, and hardly perceptible, and life itself in the act of vanishing! Strange to say, from this low ebb he rather suddenly emerged—spoke, took nourishment—and became considerably better than he had been at any period during the two preceding days. Sanguine hopes were entertained of his recovery during the 30th and 31st days of December—there being often a considerable interval between the paroxysms, during which he was cheerful and full of hopes himself!

It was after this miraculous resuscitation that we observed a phenomenon not before presented, and which did not afterwards recur. In two successive paroxysms, the face and eyes were drawn violently to the *right* side, the spasms in the extremities and trunk continuing nearly the same as usual.

The constitution, which had hitherto borne up wonderfully against such unparalleled tortures, now evidently began to give way. The features became shrunk, and expressive of unutterable suffering; and he expressed a conviction—almost a wish, that the Almighty would release him from his agonies, one way or other! The paroxysms continued, but neither so violent nor so protracted as at an earlier period of the disease:—He got some sleep in the night of the second of January, and expired towards the morning.

We have not interrupted the narrative of this terrible malady, by an enumeration of the remedies employed—for, candidly speaking, we very much doubt whether the disease was, in any material degree, even mitigated by the various means that were tried in this deplorable case. When the list of medical attendants is referred to, it will hardly be doubted that every remedy which talent or experience could suggest was fairly employed. Cupping, leeching, blistering, purging — and, lastly, *mercurialization*, were tried—and all failed. The dissection will well excuse the want of success in therapeutic agents. One feature was prominent throughout—the extreme vitiation of the fæcal evacuations. They continued of the most depraved kind till the last —or till there was nothing more than mere watery secretion to come away. Opium was not extensively employed, on account of a most decided antipathy which the patient entertained against the medicine. In early life this gentleman had studied physic, and he was able to detect at once the smallest quantity of opium, when exhibited, by its effects on his bodily and mental functions. It is very doubtful, however, if the extensive exhibition of opium, in any shape, would have been productive of any material benefit. The only remedies which seemed to promise any mitigation of the symptoms were purgatives and mercury. The former generally diminished, for a short time, the frequency and violence of the spasms :—The latter (calomel in two or three-grain doses every two or three hours) promised, at one time, a relief that engendered hopes of a happy result; but alas! these hopes were delusive, and every remedy failed in the end. The calomel could not be got to affect the mouth—and ultimately so irritated the bowels, that opiates became indispensable.

### Dissection.

It was with the greatest difficulty we got permission to examine the head. Sir Astley Cooper, Dr. Bright, Dr. Watson, Dr. Johnson, Mr. Macintyre, Mr. Balderson, and Mr. H. J. Johnson were present. The cranium was remarkably thick, especially about the os frontis, where the diploe was obliterated. The membranes covering the fore and upper part of the right hemisphere were thickened, and strongly adherent to the subjacent brain. In the falciform process, deep between the anterior lobes of the brain, and near the inferior longitudinal sinus, was found a rough and jagged bone, an inch and a quarter in length, by about half an inch in breadth, well calculated to irritate both hemispheres. In the middle of the anterior lobe of the *right* hemisphere, near its upper surface, was found a portion of brain, larger than a walnut, reduced to the consistence of a yellowish fluid, contained in a cavity lined by a polished surface, but not exhibiting the organization of a membrane, or cyst. Surrounding this cavity and its liquid contents, the medullary substance of the brain was preternaturally hard and firm, like an indurated circle, contrasting remarkably with the brain in general. The substance of the brain, immediately under the thickened and adherent membranes, was in a state of

*ramollissement*. There was considerable vascularity about the base of the brain and pons varolii, but no other morbid appearance deserving of notice.

Many circumstances, which cannot be mentioned here, combined to render this case exceedingly interesting—and not the least of these was the horrible torture experienced by the patient himself, once one of the faculty, and nearly connected with some of the first medical characters in this or in any other country. We here see an organic lesion, or rather lesions, of the brain, which must have been of several years' standing, and yet producing functional disturbance only occasionally—and often at long intervals—the health, in such intervals, being apparently good—at all events, without any impaired state of the cerebral functions. It may be a question, what part the bony deposit in the falx took in the chain of causation. We are of opinion that, in conjunction with the great thickness of the cranium, this irritating body, placed deep between the two hemispheres of the brain, must have conduced largely to the epileptic attacks with which the patient was troubled for some years previous to his last illness. The diffluent portion of brain, in the anterior lobe of the right hemisphere, must have been of some standing; but we are inclined to think that it was rather an effect than a cause of the epileptic seizures—at all events, that it took place *posterior* to the early attacks, and, consequently, could not be considered in the light of *their* cause. But that it was mainly instrumental in the fatal catastrophe we have little doubt. The convulsions were always greater in the left than in the right side—while the face, excepting in two paroxysms, was constantly drawn to the opposite side in the fits. The most inexplicable part is the circumstance of the paralysis, which generally succeeded the violent spasms of the *left* arm, but without any such event in the left side of the face. Why was the face drawn twice to the *right* side—and all the rest of the times to the opposite side? This is a puzzler? Could the bony deposite in the falx, situated *between* the two hemispheres, account for this phenomenon? Circumstances might possibly arise, in the course of the disease, that might cause the irritation to be more directed towards one hemisphere than towards another, at different periods. It is remarkable that, with such lesions, health should be but slightly impaired—and the mental functions not at all—the day before the fatal attack commenced! The disordered condition of the bowels was the only ostensible cause why the disease was called into such destructive activity. This gentleman had strong prejudices—we might say antipathies, especially against calomel, and every preparation of opium. He had also the strongest objections to many other medicines—circumstances that crippled his medical attendants in the first few days of the malady, and probably proved detrimental to the patient himself. Considering that the organic changes must have existed for a long time, it is by no means improbable that, if the digestive functions had been restored to a healthy state during the first two or three days of the spasms, the termination might have been otherwise than it was—and that a temporary respite might have been obtained.

---

### Acute Nephritis.

The following case is quoted by Dr. Ryan from a thesis on "Acute Nephritis," supported by M. Lemoine, of Paris. As we have not seen the thesis, we shall copy the case, being short, from our contemporary.

"N., aged 50 years, of plethoric constitution, had for two years passed very fine gravel with his urine, accompanied with considerable pain; the urine was reddish, the stream bifurcated, though he asserted that he had never had gonorrhœa. During the last year he has passed no gravel, but the emission of urine has been exceedingly difficult and much more painful. For some time the desire to pass water has been very frequent; the stream would suddenly stop, then go on again, and so repeatedly: the pain was excruciating. On entering the hospital calculus was suspected, and a sound was introduced; nothing was found. A few days after, the emission of urine having become next to impossible, an useless attempt was made to introduce a catheter: Ducamp's sound was then used, and a constriction five inches down the passage was found. Subsequently vain endeavours were repeatedly made to introduce bougies. On the 16th, however, they contrived to fix a bougie, which entered the bladder with the utmost ease, and without causing the slightest pain: it remained in all day. On the 17th, the patient had rigors on coming out of the bath: shortly after he was seized with colics; diarrhœa came on, and pains were felt in the penis (demulcents and anodynes). The 19th: in the same state: thirst great: urine was passed 8 or 10 times in the day, and 10 or a dozen times in the night: during the emission, and for a long time after, the urethra and rectum were acutely painful: the pulse tranquil: tongue moist: no sleep. The 20th: an acute, darting pain was felt in the right lumbar region: the belly is somewhat swelled and painful to the touch: constipation: vomiting of bilious matter: yellow tongue: intense thirst: great head-ache: small and rapid pulse (leeches on the pained part, hip-bath, demulcent drinks). From the 20th to the 24th the state of the patient was not improved: the treatment was continued. On the 24th an ounce of castor oil was given. The 25th: the pulse is steady, the vomitings had not ceased: the pain is agonizing; the belly is slightly swelled and numb in the iliac region and the whole of the left side: the face much swelled (sinapisms). Died at noon.

*Dissection.* The right kidney is surrounded with thick pus infiltrated into the surrounding cellular tissue. It is considerably larger than the left kidney, and more red: both its surfaces are spotted with white or suppurated points of the renal tissue: one on the convex edge is larger than the others. Between these purulent points, the tissue of the kidney is of a natural colour. Around those on the posterior surface there is, however, a dark, red ring: the purulent points are spongy and easily torn or broken.

Within the kidney, the membranes of the pelvis and ureter to an inch from the kidney are thickened and covered with small points, some pink, others dark red or blackish: under the latter, the mucus was particularly thickened. In all the divisions of the calices the mucus is of a dark red. All the papillæ are inflamed and exceedingly red: they are soft and easily broken by pressure with the finger. Some of them are ulcerated at the point; others are completely disorganized and pulpy. The suppuration extends along the tubular fasciculi to the external diseased points. In some papillæ the disorganization only extends to the cortical substance; in others innumerable points of ulceration are observed. The cortical substance is partly sound and whitish.

The bladder is reduced to a remarkably small size, and contracted behind the pubis; its parietes are thickened to more than half an inch. The urethra is very red, and covered with small and numerous vessels gorged with blood. The muscles of the perineum are strongly developed."*

We can hardly fail to remark the inert practice which led to the fatal catastrophe in the above case. Little doubt can be entertained, that nephritic inflammation was preying on this poor man during the whole time that was lost in catheterism, warm bathing, and the "médecine expectante." Had he been copiously bled and cupped, or leeched, often and long before the warm baths were employed, he might have had some chance of life. As it was, he had no more hope of recovery than a man thrown into the sea, with a best-bower anchor made fast to his body!

---

* Dr. Ryan's Journal, No. 153.

### PATHOLOGY OF THE URINE. By Dr. BOSTOCK.

There is a neat exposé of this subject in a late number of the Cyclopædia, the pith of which we shall give in the following extract. Previously to touching on the morbid states of the urine, Dr. B. gives the analysis of healthy urine by Dr. Henry and also by Berzelius. They differ in some respects—but not very essentially.

| | " *Henry.* | *Berzelius.* |
|---|---|---|
| 1. | Water. | Water. |
| 2. | Free phosphoric acid. | |
| 3. | Phosphate of lime. | Phosphate of lime. |
| 4. | Phosphate of magnesia. | Phosphate of magnesia. |
| 5. | Fluoric acid. | |
| 6. | Uric acid. | Uric acid. |
| 7. | Benzoic acid. | |
| 8. | Lactic (impure acetic) acid. | Free lactic acid. |
| 9. | Urea. | Urea. |
| 10. | Gelatine. | |
| 11. | Albumen. | |
| 12. | Lactate (acetate) of ammonia. | Lactate of ammonia. |
| 13. | Sulphate of potash. | Sulphate of potash. |
| 14. | Sulphate of soda. | Sulphate of soda. |
| 15. | Fluate of lime. | |
| 16. | Muriate of soda. | Chloruret of soda. |
| 17. | Muriate of ammonia. | Chloruret of ammonia. |
| 18. | Phosphate of soda. | Phosphate of soda. |
| 19. | Phosphate of ammonia. | Biphosphate of ammonia. |
| 20. | Sulphur. | |
| 21. | Silica. | Silex. |
| | | Extract of meat soluble in alcohol. |
| | | Extractive matters soluble only in water. |
| | | Mucus of the bladder." |

The morbid states of the urine are divided into six kinds—and as the subject is handled in a masterly manner, and the language cannot be condensed without injury, we shall make room for a long extract, in order not to break the continuity of the pathology.

1. *Aqueous Urine.*—This may be characterized as a state of the secretion, where the proportion of the fluid to the solid contents is morbidly increased, and which is frequently, although not necessarily, attended by an increased quantity of the urine that is discharged. And here we must be careful not to confound those incidental or temporary changes in the state of the fluid, such as are produced by the application of cold to the surface of the body, or by certain mental emotions, with the watery urine, which proceeds from a permanent or constitutional cause. Among the causes of this description, perhaps the most frequent and the most efficient is that state of the system which has been denominated the nervous temperament, and more especially the peculiar modification of it which gives rise to the hysteric paroxysm. Here we have an unusually large quantity of urine discharged, and this often containing less than the ordinary proportion of solid matter, and as far as has been ascertained, without any other essential change in the nature or proportion of its constituents. The aqueous urine will be often found to exist in that state of the system, where, without any specific disease, the powers both of the mind and the body begin to decline, and when the gradual decay of all the organs indicates the approach of old age.* Here the functions of the kidney are among the first to feel the effects of that decay, which gradually undermines every part of the system, and which terminates in its dissolution. We are not aware of any specific cause

* " The late Dr. Heberden, in his invaluable volume, enumerates the frequent passing of urine among the symptoms of what he styles Valetudo Conquassata; Comment. chap. 94; in this state we believe the urine will be found to be much increased in quantity, and to be aqueous."

of this particular condition of the urine, independently of the general irritability and debility of the system; nor do we know that it leads to any specific indication either of prognosis or of treatment. We believe, however, that it is an actual and not a very unfrequent occurrence, and that it is one to which the attention of the practitioner may be advantageously directed, as it may assist him in forming an opinion respecting the general state of the vital energies of the system, and of the degree in which the organs of digestion and assimilation are capable of performing their functions.

2. *Sub-aqueous urine.*—This, in its specific character and its appellation, may be considered as directly the reverse of the former state, and yet, in its pathological cause, is somewhat closely connected with it. In certain cases of increased discharge of urine, it will be found, that besides an augmentation in the quantity of the secretion, it contains even more than the ordinary proportion of solid contents, and thus, both fiom its quantity and its quality, carries off from the system an unusually large proportion of the ingredients, which ought to be applied to its growth or support. The sub-aqueous urine is sometimes found to exist in the decline of life, and would appear to be one cause among others by which a premature state of decay is effected. But it more frequently occurs at an earlier period in constitutions that have been debilitated by various causes, and particularly by excesses of all kinds; by long-continued violent exercise, by stimulating diet, and especially by large potations of wine and fermented liquors, and perhaps, more than any other cause, by excessive sexual indulgence. This state of the urine is occasionally met with in persons whose constitutions have been injured by a residence in tropical climates.

The sub-aqueous urine is often found to be connected with the albuminous, or to alternate with it; and it would appear to be, to a certain extent, the result of the same causes acting upon a different constitution, and exhibiting themselves in a more chronic form, consequently with less of febrile action and without the specific derangement of any particular organ. To this species we are disposed to refer the variety of diabetes which has been termed *insipidus*, where we have an increased flow of urine, of high specific gravity, with the constitutional symptoms of the disease, both as to the state of the digestive organs and the skin, but where the urine does not contain any saccharine matter.* We believe it will be found upon examination that the sub-aqueous state of the urine is not unfrequently a precursor of its saccharine state, and that, in some cases, which may be correctly styled diabetes, the disease never proceeds beyond this stage, in consequence of some change being effected in the constitution, either by natural causes or by the operation of remedies.

3. *Lithic urine.*—This is characterised by the spontaneous deposition of lithic acid, constituting the principal part of what is generally termed the sand or gravel of urine. The immediate cause of this deposition is the presence either of some other acid in the urine, which may precipitate the lithic acid naturally contained in it, or merely an increased quantity of the lithic acid itself, or rather the super-lithate of ammonia, so as to be more than can be held in solution in the urine, especially when it is reduced in temperature, after being discharged from the bladder. It appears that this latter, which is the simplest form of the disease, is also the most frequent, and in fact that there are few individuals in whom it does not occa-

---

* "The writer of this article presented a case of this description to the Medico-Chirurgical Society, where the urine contained no saccharine matter, but where its specific gravity was 1034, while the quantity discharged daily amounted to five quarts. By an analysis of the urine it appeared that the patient was discharging daily seven and a half ounces of urea, which may be considered as at least three times the natural quantity. Med. Chir. Trans. vol. iii. p. 107 and seq."

sionally exist:* we find, indeed, that any cause which deranges the digestive process, or interferes with it due action, may produce the deposit of lithic acid. Among the most frequent of these causes we may enumerate an excessive quantity of food taken into the stomach, food of an indigestible nature, exercise taken immediately after a full meal, the application of cold to the surface of the body or to the feet during digestion, and an undue quantity of liquid taken into the stomach.

In the most simple form of the disease there is a deposit from the urine, as it cools, of a brown sediment, which is either pulverulent, or consisting of a mixture of the powder with minute crystalline spiculæ. When this deposit, as is frequently the case, assumes a pink or purple colour, it depends upon a combination of the purpuric with the lithic state of the urine. In the less simple form of the disease there would appear to be an additional agent in the urine, besides the superabundant lithic acid, which produces the deposit in question. Dr. Prout supposes that the precipitation may be effected by various acids, but most generally by the phosphoric, and occasionally by the sulphuric, the nitric, or the carbonic, as well as by some other acids which are the peculiar product of the renal secretion.† In this case the lithic acid is precipitated in more or less of a crystalline state, and forms the greatest part of what has been termed urinary gravel. This gravel, like the sand in the former variety, may be tinged of a pink or purple colour, depending, in like manner, upon the combination of the purpuric with the lithic urine.‡

With respect to the pathology of the lithic urine, it may be generally considered as a symptom of an imperfect state of the digestive organs, either induced, in each individual case, by a specific cause acting upon the stomach and the chylopoietic viscera, or by a peculiar condition of the constitution, more especially by the gouty diathesis.§ It is this state of the urine which lays the foundation for a large proportion of the calculi which are occasionally formed in the bladder, or other parts of the urinary organs. We find, by chemical analysis, that the most frequent species of these concretions consist essentially of lithic acid, and that in many others, where the bulk of the calculus is composed of the phosphates, the nucleus consists of lithic acid.|| (See CALCULUS.)

---

* Prout's Inquiry, p. 121.

† Inquiry, p. 128.

‡ " For a very complete account of this variety of the urine, and of the state of the system which produces it, the reader is referred to the valuable treatise of Dr. Philip, first published separately in the year 1792, and afterwards, in an altered form, in the sixth volume of the Transactions of the College of Physicians, and to the third chapter of the second section of Dr. Prout's Inquiry. Both these writers refer to a work, published nearly half a century ago by Forbes, which appears to possess very considerable merit, when we consider the imperfect state of chemical science at that period."

§ " We are indebted to Wollaston for the discovery of the chemical nature of the gouty concretions, which had been previously termed chalk-stones, as consisting of the lithate of soda. Phil. Trans. for 1797, p. 389. These bodies had been previously examined by Berthollet; but it appears that this eminent chemist, who on most occasions is so remarkable for his accuracy, in this instance proceeded rather upon theory than actual experiment. He supposed that the urine of gouty patients was deficient in phosphoric acid, and that the acid was deposited in the joints in combination with lime. Journ. Phys. t. xxviii. p. 275. In connexion with the subject of gouty urine we may refer to the work of Sir C. Scudamore, who performed a series of experiments on this subject."

|| " *Fourcroy and Vauquelin*, Ann. Chim. t. xxxii, p. 217; Philip, Trans. Coll. Phys. v. vi. p. 176; *Pearson*, in Phil. Trans. for 1798, p. 38; *Marcet*,

4. *Phosphatic urine.*—This, when considered in its chemical relations, may be regarded as the opposite state to the lithic, consisting essentially in an excess of the phosphoric salts, and especially of the phosphates of lime and of magnesia, with a comparative deficiency of the lithic acid. Hence it is characterised by the deposition of the phosphates in the form of a sediment, which is generally white, and of an earthy or chalky appearance, although occasionally assuming the crystalline state.—The chemical constitution of the phosphatic urine appears to differ considerably in different cases, all of which, however, agree in the characteristic circumstance of the excess of the phosphoric salts. Sometimes there is a deficiency of the urine in proportion to the saline ingredients, and at other times there is an absolute deficiency of both the urea and the salts, reducing the urine to the aqueous state, but still maintaining the relative excess of the phosphates.

The pathological condition of the system which produces the phosphatic urine is less easily characterised than the lithic, as it would appear that a variety of circumstances, which have no very obvious connexion with each other, both of a constitutional and a local nature, agree in producing this morbid change in the state of the urine. The phosphatic urine is frequently observed in sickly and ill-fed children, in those that inherit a scrofulous constitution, or where we have reason to suspect the existence of diseased mesenteric glands. Again, in the decline of life we find that various circumstances, which contribute to break down the constitution, and affect the digestive and assimilative functions, diseases of the glandular system, and especially local injuries of the parts contiguous to the kidney, are among the frequent causes or concomitants of the phosphatic urine. It would appear also to be produced by mechanical irritation of the bladder; for it has been observed that a considerable number of the calculi, which are principally composed of the phosphates, possess a nucleus of lithic acid, or some substance which has been accidentally introduced into the bladder, and which appears, by its action upon that organ, to have contributed to the production of the excess of the phosphates, and their consequent deposition. As in the case of the lithic urine, so the phosphatic is sometimes connected with the purpuric state, thus affording deposits of various shades of colour.* We also find that the lithic and the phosphatic urine not unfrequently alternate with each other: it is this state of things which gives rise to one of the species of calculi which have been termed, from their mechanical formation, alternating.† Upon the whole, we must consider the phosphatic urine as indicating a greater derangement of the system, and one which is less under the influence of curative means, than the lithic.‡

5. *Purpuric urine.*—This is characterised by the colour of its deposit, which, in its ordinary state, has obtained the name of the lateritious sediment. It was recognized as the indication and concomitant of the febrile state of the constitution, among the earliest observations that were made on this fluid, and one which has always been regarded as among the most de-

---

ch. v.; Prout's Inq. p. 95 et seq.; *Henry*, Med. Chir. Trans. v. x. p. 132; *Smith*, Med. Chir. Trans. v. xi. p. 10; *Egan*, in Tilloch's Phil. Mag. v. xxiii. p. 199 et seq."

* "The combination of the phosphatic and the purpuric deposits may in most cases be distinguished from that of the lithic and the purpuric, by the latter being tinged with the brown colour of the lithic acid, while the former, being a mixture of a white and a purple substance, are of a purer pink colour."

† Marcet, pp. 59, 96.

‡ "Perhaps one of the most valuable parts of Dr. Prout's work is his chapter on what he styles 'the phosphatic or alkaline diathesis;' although, were we inclined to be hypercritical, we might object to the term *alkaline* as applied to this state of the urine."

cisive pathognomonic symptoms of an increased action of the arterial system. The first attempt to ascertain the immediate cause of the change of the urine appears to have been made by Proust,* but not with any great success, as he does not appear either to have ascertained the exact nature of the change, or the means by which it is effected. For the more correct information which we possess on the subject, we are indebted to Dr. Prout, who investigated it with his accustomed acuteness, and proved that a new substance, possessing the properties of an acid, is produced in the urine, which, from the colour of its combinations with the alkaline bases, he termed the purpuric acid.† He also rendered it probable that all the shades of colour which were observed in the urinary deposits, from reddish brown to pink and purple, may be referred to the mixture of the purpuric with the lithic acid, or with the phosphates of lime and magnesia.

With respect to the pathology of the purpuric urine, it may be simply characterized as the urine produced by that increased action of the arterial system which constitutes inflammatory fever; which, therefore, may occur either in its simple state, or in combination with the lithic or the phosphatic urine, when any circumstance induces febrile action while either of these states is present. It has been supposed that the purpuric urine is more immediately connected with the diseases of certain of the abdominal viscera, but we conceive there is reason to doubt whether this opinion be tenable, and we undoubtedly find that the purpuric urine exists, in the most marked form, where we have been unable to discover any traces of local inflammatory action. The existence of the purpuric urine in its various shades and combinations is almost as frequent an occurrence as the lithic, and is in many cases produced by very slight derangements of the system. But when, on the other hand, it exists in a high degree, and continues for a long time without interruption, it indicates a morbid derangement of the functions, which it is often difficult to remove, and which must be regarded as leading to an unfavourable prognosis.

6. *Albuminous urine* is distinguished by its exhibiting the presence of the proximate principle from which it derives its specific appellation, when the appropriate tests are applied to it. The albumen sometimes exists in so great a quantity as to render the fluid more or less opaque when it is discharged from the bladder; but it may be frequently detected in the fluid by exposing it to heat or to certain chemical re-agents, when it is not otherwise visible. It seems to have been first distinctly brought into view by Cruickshank, in the essay to which we have referred above:‡ it was afterwards very carefully described by Blackall, who considered it as a pathognomonic symptom of a peculiar species of dropsy, which, as he conceived, required a specific mode of treatment,§ and has more lately been made the subject of consideration by Dr. Bright, in the series of his valuable pathological observations.‖ So ample an account of these observations, and of the discussions connected with them, has been given in the former parts of this work, and more especially in the article DROPSY, that it will be necessary for us to do little more in this place than to refer to them. We shall only remark that the

* Ann. Chim. t. xxxvi. p. 265-9.

† Phil. Trans. for 1818, p. 420 et seq., and Med. Chirurg. Trans. vol. ix. p. 481, 2.

‡ Rollo on Diabetes, and Tilloch's Phil. Mag. vol. ii. p, 248.

§ " Observations on Dropsy. We have a judicious critique on this work in the Edin. Med. Journ. vol. ix. p. 334 et seq."

‖ " In connexion with the account of the albuminous urine, which is contained in Dr. Bright's Medical Reports, the reader is referred to Dr. Christison's remarks in the Edin. Med. Journ. vol. xxx. p. 107 et seq. and to the observations of Dr. Gregory in vol. xxxix. p. 54 et seq."

albuminous urine is symptomatic of increased action of the arterial system, connected with visceral derangement; generally of the kidney, (when it is frequently attended by dropsy) and occasionally of the liver, so as to constitute a very formidable disease. In other instances, however, it would appear to be produced simply by a morbid condition of the digestive organs, unconnected with any structural disease, and in this case to be of transient occurrence, and to be indicative of a slight derangement of the functions.

7. *Saccharine urine*, like the albuminous, derives its specific name from the presence of the proximate principle which is found to exist in it, and which constitutes the most remarkable pathognomonic symptom of the ordinary form of diabetes. This disease had been very correctly described by the ancients; but although they noticed the increased flow of urine, its peculiar condition, as containing a quantity of saccharine matter, was first pointed out by Willis. Of late years, since the chemical constitution of the animal fluids, both in their healthy and their morbid states, has been more attended to and better understood, the saccharine urine of diabetes has been the subject of very numerous experiments. The result appears to be, that the urea is in a great measure deficient, while in its place the kidney secretes a substance which has every property of sugar, both as ascertained by the spontaneous changes which it undergoes, and by the action of chemical re-agents upon it.*

With respect to the pathology of the saccharine urine, we may make the same remark as in the last section, that it has been so fully and ably discussed in the former part of this work in the account of DIABETES, that nothing remains for us but to refer our readers to that article, where they will find detailed every circumstance of importance connected with the peculiar state of the urine, the symptoms and prognosis of the disease, and its treatment. The only point on which we feel it necessary to offer any remark respects the nosological question, whether it be proper to confine the term diabetes to that state where the urine contains sugar, and which has been styled the diabetes mellitus. We have already given it as our opinion, that we believe a state of the constitution exists, which is intimately connected with the diabetes mellitus, and is indeed frequently the commencement or first stage of it, where we have every symptom of the disease, both general and local, except the saccharine state of the urine. As a question of technical nosology, it may be of little importance; but we are disposed to think that, if the practitioner were aware of the possibility of such an occurrence, he might perhaps, in some cases, be able to check the disease at the outset, and prevent it from acquiring the saccharine state, when it becomes so formidable in its symptoms, and so exceedingly difficult to remove."

There is an eighth section, devoted to miscellaneous or incidental circumstances, on which we shall not dwell. Dr. Bostock would have made the above article much more complete, and much more useful to the great mass of his readers, if he had added a concise account of the modes of applying the chemical tests to the analysis of urine. Authors, and especially chemical writers, should not conclude that all their readers are as well acquainted with the details of chemical analysis of the fluids as themselves. The modes of applying the tests to unhealthy urine are far from being generally known—and still less are they generally practised. The state of the urine, indeed, is too much neglected, even by talented and experienced practitioners.

---

* "In the eighth volume of the Med. Chir. Trans. p. 537, Dr. Prout has given the analysis of the saccharine urine: see also Chevreul, Ann. Chim. t. xcv. p. 319, and Bostock's Physiol. vol. ii. p. 361."

### Peculiar Affection of the Lungs of Infants.

M. Joerg, of Leipsic, has written an inaugural dissertation on this peculiar affection, from which a short extract has been made in one of our weekly contemporaries. It runs as follows:

" After some remarks on the physiology of the fœtus and the new-born infant, the author enters upon the description of the disease, and at once specifies its nature as consisting in a *concretion or agglutination of the pulmonary cells,* occupying a varied extent of one or both sides of the lungs. This agglutination does not proceed from inflammation, nor effusion, but from an imperfect respiration, which prevents the air from penetrating all the cells: the parts thus affected, therefore, retain the colour and consistence of the lungs of the fœtus, and do not swim in water.

The causes of this affection, a too easy and too quickly finished labour, and a too strong compression of the head of the child during delivery. These two circumstances prevent the respiration from being as full as it ought to be from the very first period, and thus give rise to the disease in question. This disease is particularly characterized by a superficial, short, anxious, and scarcely perceptible, and sometimes intermittent respiration, a feeble, plaintive voice, difficulty of sucking, a shortened elevation of the sternum and ribs, livid or blue colour of the skin, coldness of the body, universal debility, weak and slow pulse. These disorders of the respiration and circulation occasion imperfect nutrition, congestion of the brain, convulsions, and, in consequence of the violent respiratory efforts, inflammation of the bronchi and lungs.

So long as it is possible to make the child take a deep inspiration it is possible to save it. But if the respiration continues weak in spite of our endeavours, the patient is lost. The treatment consists in clearing the mouth of the mucus in it, striking the soles of the feet and the palms of the hands, as also the chest and back, with rods; rubbing the chest and back with sulphuric ether, and introducing it into the nostrils and mouth. If the voice and respiration continue weak, these means should be persevered in during the time the child is in a simple or aromatic warm bath. Besides these means, repeated clysters and emetics are employed; the latter, however, and indeed all means that drive the blood towards the head are contra-indicated when the brain is injured; in these cases calomel and sinapisms are useful by establishing a revulsion on the intestinal mucous membrane and skin, withdrawing the blood from the head, and preventing the excessive secretion of mucus in the bronchi. Moderate inflation of the lungs with warm air is also advisable; the introduction of oxygen or pure air containing too great a concentration of oxygen (*cold* air we presume the author means) is not advisable, the lungs of a new-born child being too delicate for such stimulation. Nine cases are appended to Dr. Joerg's work; his researches will doubtlessly contribute to increase of knowledge of the pathology of new-born infants, which is still so obscure."*

---

### Is there any Connexion, and what, between Acute and Chronic Rheumatism?

This question excited some surprize in the Westminster Medical Society, at a late sitting, and was warmly discussed. It was maintained by Dr. Johnson, that the two diseases above-mentioned had no necessary connexion—that they were different in their seat, their nature, and their symptoms, as well as in their cure. He contended that the seat of chronic rheumatism was in the *muscular* fibres or their investing membranes—while that of acute rheumatism was in the synovial and ligamentous—or in other words, in the white fibrous structures. For all practical purposes, he observed, it was sufficient to say that the *acute* disease was in the

* Dr. Ryan's Journal, No. 153.

joints and immediate neighbourhood, while the *chronic* affection was between the joints—that is to say, in the muscles.—Again, the one was attended with inflammation and fever, as well as pain —the other unattended by inflammation or fever. Chronic rheumatism was not the sequela of acute rheumatism. For one instance of chronic rheumatism succeeding acute, there are five hundred where there has been no preceding rheumatic fever. We do not see the two diseases become converted into one another, as we would, if they were only grades or conditions of the same affection. Acute rheumatism travels from joint to joint, and frequently is translated to internal organs —especially to the heart. Chronic rheumatism never affects the heart—and does not shift its place from muscle to muscle, as those who have experienced its tenacity to its ordinary locality can testify, to their cost. It is unnecessary to dwell on the difference of treatment, and the etiology of both is involved in obscurity. The one complaint appears to be a specific phlegmasia—the other a specific neuralgia. Acute rheumatism may be, and often is, followed by a chronic complaint—*in the same parts* —the synovial and white fibrous tissues—but not by chronic rheumatism in the muscles.

All these considerations, and many others that might be mentioned, induced Dr. Johnson to reject the term "acute rheumatism," and "rheumatic fever," entirely, and to substitute Arthritis—a term to which it is as much entitled as gout. There is no essential diagnostic mark or difference, he maintained, between gout and acute rheumatism—and, therefore, they were but modifications of the same disease, depending on idiosyncrasy, and other circumstances with which we are unacquainted. It was objected to Dr. Johnson, that acute rheumatism was almost confined to the poor, while gout was almost exclusively the portion of the rich. It was also stated, that gout was greatly dependent on disordered conditions of the digestive organs. These arguments, Dr. J. contended, were in his favour. There was as much disorder of the digestive organs among the poor as among the rich—if not more. The difference of circumstances in which the two classes are placed, might cause the difference between gout and acute rheumatism. All practitioners acknowledge that, in many cases, they cannot tell which is the disease—and, therefore, frequently adopt the terms "rheumatic-gout," or "gouty rheumatism," which the community have long before adopted. Neither is there any very material difference in the treatment of the two modifications. In both, there is occasional necessity for local depletion—and in neither is there often occasion to bleed from the arm. Colchicum is as useful in the one case as in the other. In both, the urinary secretion is high-coloured and lateritious. In both, the digestive organs are in fault. In both diseases, the inflammation is prone to travel from place to place, and to attack internal organs. In both, it is unsafe to apply cold topically to the inflamed part—especially where the constitution is not vigorous, and where all the internal organs are not performing well their functions. Finally, Dr. J. contended that, both in gout and acute rheumatism, the warm bath is seldom safe—except where there is a metastasis to an internal organ from the extremities.

These hints are thrown out for the consideration of our brethren.

---

### Remarkable Case of Diseased Heart.

The case here narrated occurred in St. George's Hospital, under the care of Dr. Seymour, and was communicated to our weekly contemporary, conducted by Dr. Ryan. It is as follows :

"A man named Lane was admitted about six weeks since under the medical care of Dr. Seymour, labouring under pulmonary bronchitis, with some obscure symptoms of affection of the heart. The usual remedies were administered with very beneficial effect, which, however, soon passed off, and the symptoms continually recurred.

The patient coughed up some frothy mucus, tinged with bloody sputa, but nothing more. We should also remark that there was palpitation of the heart, and that the remedy from which his general symptoms derived the greatest relief was calomel and opium.

His symptoms proceeded in this manner towards a gradual improvement for some time, when, having occasion to go to the water-closet, and strain at stool, a most intense spasmodic paroxysm of pain came on violently, shaking and convulsing all the muscles of the body, and requiring four men to control him. Coupled with this, was very great and severe dyspnœa, which shewed itself in his features so strongly, as amply to justify the truth of Dr. Seymour's remark, that he had never seen a countenance indicative of such intense agony. He complained, however, most of severe and excruciating pains in both lower extremities. Within an hour after this paroxysm gangrene supervened in the right lower extremity, and the usual remedies of warmth and stimuli were administered, the limb was wrapped round and kept warm by means of hot flannels. The actual pulse could not be felt in the right inguinal region for two or three days previous to his death.

We attended the post-mortem examination on the 19th. We understand that the man expressly wished that his body might be examined after death. The general cellular structure of the lungs was engorged with blood, but not sufficiently so as of itself to form a cause of death; the right lung was, however, more vascular than the left, and on its anterior external surface there was a spot, of about the size of a sixpence, which presented a darker and more gangrenous appearance than the surrounding surface, and which was in a state which, for want of a better name, we shall denominate apoplectic; there was also some serum effused on the same side, and some marks of old adhesive inflammation could be seen. On the surface of the pericardium being exposed, it appeared to be unusually large, and on being slit open, the heart was found to be greatly enlarged. As, from the symptoms during the latter period of the patient's life, the diagnosis formed was that of aortic aneurism, the heart with the aorta, as far as the termination of the common iliac arteries, were removed, but no aneurism could be detected. There was a dark, gangrenous spot over the middle of the anterior and external surface of the right ventricle, which Dr. Seymour believed would reveal something of the mysterious cause of all the symptoms under which the man laboured; this prognosis was not however verified. On cutting open the heart, its cavities were found to be unusually enlarged, and its walls proportionally thinned, and at the base of the right ventricle the structure of its walls was found to be completely disorganized; it crepitated upon pressure between the fingers, and presented the appearance, as Dr. Seymour very justly remarked, of a muscle after it has been severely bruised, the other portions of the heart presented no other unusual appearances than those we have mentioned; on slitting open the aorta, its lining membrane was found to be unusually vascular, and studded here and there with patches of atheromatous matter, at one portion of it, (the anatomical relation of which we cannot state, from the parts being isolated when we saw them) the cellular tissue beneath the lining membrane appeared broken down and disorganized, the membrane itself over it being entire. At its bifurcation the right common iliac was plugged up with coagulated blood, an appearance which we also remarked was presented along the femoral artery of the same side, which Sir Benjamin Brodie laid open through the entire course. The abdominal viscera were not minutely examined, but their general appearance indicated no great abnormal state of parts. The cranium was not opened nor its contents examined, no symptoms of any kind during the man's life being referable to any of the nervous structures.

Thus far to our minds, and to the concurrent opinion of all who witnessed the autopsy of this case, did the post-mortem appearances, detailed above, reveal nought of a satisfactory nature, at all conclusive as to the immediate exciting cause of the rapid supervention of gangrene the few days preceding the man's death. Such symptoms have generally been considered as diagnostic of the presence of an aneurism of the aorta, or some of its large iliac branches, and that extreme pains in the lower extremities has been generally caused by the bursting of the aneurismal sac, symptoms which occurred in a case admitted into the hospital, so far back as during the assistant-surgeoncy of Sir Benjamin Brodie, in which there was found, after death, an aneurismal sac, connected with one of the common iliac vessels under Poupart's ligament, as related by that gentleman to Dr. Seymour during the above autopsy.

The conclusion at which Dr. Seymour arrived, respecting the above case, was, we believe, that during the straining at stool, above referred to, some portion of the musculo-ventricular structure of the heart gave way, which so materially weakened its power of contraction, as to unfit it for propelling the blood in a continued circulatory stream into the lower extremities, and that from the circumstance of there being thus, as it were, a pause in the powers of the circulation, coupled with the fact of this patient having an old inguinal hernia of the right side, which pressed upon the vessels in this spot, the current of the blood in this limb had, thereby, a greater tendency towards coagulation afforded to it, which effectually prevented the further flow of warm blood through the limb, and thus became the cause of the supervention of gangrene in the part, and of the ultimate death of the patient.

The case, we believe, was watched with great interest by many gentlemen attending the medical practice of the hospital, who are profoundly versed in the acoustic mysteries of the stethoscope; some went even so far as to state, to a hair's breadth, the extent of the diseased surface affecting the valves of the heart; but we regretted to find that, from what we witnessed, their enthusiastic predictions were found to be in nowise verified."*

* Dr. Ryan's Journal, Jan. 3, 1835.

We are inclined to take the same view of the pathology of this case—or rather the cause of the phenomena preceding death—that Dr. Seymour has done. We may remark, however, that mortification of the lower extremities is not a very rare occurrence in hypertrophy of the heart. We have seen several instances of it. The wonder is that it does not take place more frequently. We know that great œdema of the legs and feet is almost constantly present in the advanced stage of cardiac hypertrophy, and the occurrence of sphacelus in certain constitutions, under such circumstances, is an event that might be reasonably expected—and does occasionally supervene.

We apprehend that the sneer at auscultation at the close of the narrative, is rather misplaced, and probably unjust. We do not believe that any person, however "profoundly versed in the acoustic mysteries of the stethoscope," would venture to predict "to a hair's breadth," the extent of disease in the valves or in any other part of the heart. We venture to affirm, nevertheless, that the auscultation, in this case, did prophesy hypertrophy of the heart, before the fatal train of symptoms set in, though the straining at the water-closet may have accelerated the death of the individual—*Eds.*

---

### Medical Jurisprudence.—Poisoning by Arsenic.

One of the most remarkable instances of *retributive justice* on record, has lately occurred at Bristol, and we may proudly assert that the crime of murder has been brought to light almost entirely through the instrumentality of medico-legal science. We shall greatly abridge the circumstantial evidence, in order to give ample space to the chemical and pathological portions of the trial.

An aged lady, Clara Ann Smith, who had accumulated some money, and was of penurious habits, went to lodge with a Mrs. Burdock, in Trinity St., Bristol. Having got some cold, she became unwell, and was attended by a little girl in her lodgings, who turned out to be a material witness in the end. In the course of the old lady's illness her landlady (Mrs. Burdock) administered to her a bason of gruel, which the girl observed to be of a brownish colour, soon after taking which, she vomited, and complained of dreadful pains. She spat up some blood, but did not cough. No medical assistance was summoned, and the old lady died in the course of the night. She was very privately interred, and none of her relations were then made acquainted with the death. Fourteen or fifteen months had passed away, when suspicions were excited, and, on application to the magistrates, exhumation was ordered, and an inquest was held on the body. We shall quote the medical and chemical evidence from the Medical Gazette of January 10th, 1835.

"Dr. Henry Riley stated that he went with Mr. Kelson and others to open the body of a female interred in St. Augustine's church-yard. On the coffin-plate, which was partially destroyed by rust, was written 'Mrs. Smith,' aged 60 odd years; the grave was much deeper than usual for so common a coffin, and there was no water at the bottom; the coffin was made of common elm, such as is used for burials of paupers, and was screwed down in the usual way. On the lid being removed by the undertaker, they saw the remains of a human being; there was some loose earth on the breast and abdomen, which had got in through a slit in the lid, and a considerable quantity of water, which covered part of the breast, the abdomen, legs, and arms; the upper surface of the face and neck were above the level of the water; the body had on a shroud, an under garment, a pair of stockings, marked with red thread C. S., and the remains of a cap could be traced on the head. The body-clothes were slit down the centre, so as to throw the dirt which had fallen through the opening of the lid on either side; by these means the body was exposed to view; it was that of a thin person; the chest had not fallen in, and there was no appearance of its having been opened. Mr. Kelson and witness then opened the chest and abdomen, by cutting through the cartilages of the chest and ribs on either side of the sternum, continuing the incisions downwards; thus the chest and abdomen became fully exposed. They found that all the parts which were below the level of the water were turned into adipocire; the external surface of the stomach and intestines appeared of a pale bluish colour; there did not appear any traces of disease or inflammation of the peritoneal surface of the abdomen; he did not think that there was any water in the latter cavity until it flowed into the thorax, and thence into the abdomen, at the time of opening; they next took out the whole of the intestinal canal from the œsophagus downwards, and placed the different parts in separate vessels; in separating the small intestines from the duodenum, they noticed a considerable quantity of a yellow substance covering the mucous membrane of the latter, and were surprised to find that the whole of this canal presented such an extraordinary degree of firmness, and was so slightly decomposed; it was as firm as that of persons who died in an ordinary way, and who have been dead but a few days; the liver had shrunk to a fourth or fifth of its natural size; it was not thicker than his hand; the pancreas presented nothing peculiar in its appearance; the lungs had collapsed, and were reduced to a fourth of their volume; they were of a dark colour, approaching blue or grey; he did not see any thing like tubercles in them, nor any thing peculiar with regard to the pleura, nor did he observe any adhesions; the heart was collapsed and shrunk, but there was no appearance of disease in it; the diaphragm was little changed and perfect. Witness cut off the head, for the purpose of identity by the teeth. The stomach and alimentary canal were removed to the Medical School for examination; there were yellow spots in several parts of the peritoneum; they were of a bright yellow colour, identical with that alluded to as being seen on the mucous membrane of the duodenum; they were in the largest quantity on the stomach, and also visible on the upper part of the intestines, and were besides visible in one or two places on the mesentery; where the spots were seen outside, they were also traced internally, as extending through the whole substance of the intestines; the mucous membrane of the stomach was firm to an extraordinary degree, and there were no ulcerations upon it; the dark red colour it presented might either proceed from intense inflammation or from decomposition; he inclined, however, to the opinion that it was the former, otherwise the membrane could not have been so firm; the greater portion of the spots were near the pyloric extremity of the stomach; the yellow matter was spread over the whole mucous membrane of the stomach as well as the duodenum; it could be scraped off in large quantities, and especially from the stomach; the matter most resembled in colour sulphuret of arsenic, commonly called orpiment; he did not think that it arose from an infiltration of bile; the mucous membrane of the jejunum presented the same dark colour; it was equally firm with the other parts; traces of the yellow matter were also seen in this portion of the intestine; in the great intestine the mucous membrane was of a very dark red, it was extremely firm, and spotted of a dark slate colour, particularly so in the neighbourhood of its upper part of the near ilio-cœcal valve; it had no yellow spots or ulcerations; the appearances of this intestine were such as are commonly found in cases of chronic diarrhœa of long standing; the slight change it had undergone was as striking as that of the stomach; in the latter and duodenum he saw little else but this yellow substance; in the smaller intestines there was a small quantity of brownish fluid, something like cocoa. He attributed the firmness of the intestinal canal to some antiseptic substance, and sulphuret of arsenic was said to be so in a very great degree; he did not mean to say that there were no tubercles in the lungs, but merely that he did not see them; the dark slate colour of the mucous membrane of the intestinal canal is said by the French pathologists to indicate chronic inflammation.

Cross-examined by Mr. Payne, (counsel for the defence.)—A chronic inflammation would certainly occasion death, and it might have occasioned a griping pain in the side. Witness had not seen much of the effects of arsenic on the human body, that is, to the extent of death; the sulphuret has the same general effects as the white oxide of arsenic; the old opinion was that they did corrode, and the modern one that they do not; he was convinced that the yellow spots on the stomach penetrated from the inside to the outside; witness has never seen a person killed by taking arsenic; it was used in cases of cancer, and some others, as cutaneous diseases, fevers, &c.; it was never administered in this country as orpiment, but when combined with an alkali, and it was then called Fowler's solution. This was the first stomach poisoned to death by arsenic that he had seen; none but a rogue or a madman would administer arsenic in so large a quantity as had been found in this stomach by Mr. Herapath; if any medical man had done it, he would deserve to be hanged. In administering Fowler's solution they proceeded with the greatest care; if arsenic were exhibited, they would expect the appearances found in this old lady's stomach, and he felt as confident as that he sat there that her death was occasioned by arsenic; the grave in which she was interred was a deep one for so poor a coffin; in some cases graves were eight or nine feet deep, and from the number buried in them the last body did not probably lie more than two feet from the surface—a very unhealthy and abominable practice.

Dr. Riley was, in a subsequent part of the proceedings, recalled, when he described the effects of Fowler's solution as distinguished from the orpiment that had been administered in this case; the effect of arsenic when taken in overdoses, would be an inflammation of the mucous membrane of the intestinal canal, accompanied with great emaciation and debility, loss of hair, and occasionally of the nails. He had heard the evidence of the girl, Allen, and his opinion was that the deceased had been labouring under a most violent ptyalism, because the membrane of her mouth had been stated to be swelled, ulcerated, and bled extremely; she spit blood, and her cheeks and lips were swelled; he was not aware that ptyalism was the result of an exhibition of arsenic; he did not mean that it would not produce ptyalism, but he had not seen it; he thought it most likely that the ptyalism was brought on by taking mercury; he did not doubt it the least in the world that arsenic produced death in the present instance. He never saw a case of death occasioned by arsenic in small doses, taken as Fowler's solution; has seen symptoms produced by it short of the loss of hair and nails, such as sickness and loss of appetite; as arsenic becomes oxidized, it is hurtful; the white oxide is used extensively to preserve birds.

*Chemical Evidence.*

WILLIAM HERAPATH.—Is Lecturer on Chemistry and Chemical Toxicology at the Bristol Medical School; was applied to on Monday last, the 22d, to examine the body of the deceased; undertook to do so, and to analyse the contents of the stomach; was present during the whole of the time of the disinterment, and the taking out of the viscera in St. Augustine's Church-yard; received them from the hands of Dr. Riley and Mr. Kelson, the medical gentlemen employed; placed them separately in two basins, which had been carefully wiped clean by himself—the stomach and duodenum in one, and the intestinal canal in the other; the stomach was whole, there was no orifice, and no loss of any of its contents worth noticing; tied up the basins with their contents in a cloth, and gave them to a man to carry to the Medical School; never lost sight of him the whole way. The appearance of the body in the church-yard was such as to lead witness to believe that it had been under the influence of an antiseptic; the body was, generally speaking, rapidly passing to a state of animal soap; or adipocire; the stomach and intestinal canal were in an unusual state of preservation, so much so, in fact, that upon seeing the viscera opened, he exclaimed, 'This looks like the effects of arsenic;' as it is a well-known fact that that poison has the effect of preserving the parts contiguous to it; and tends to assist the changing the other parts into adipocire. Upon receiving the parts, witness invited all those who were interested in the case to accompany him, as he intended to operate in public; the Solicitor, and three medical gentlemen employed by the accused, accompanied witness, with many others, to his own theatre, at the Medical School, where he put a new deal board on the lecturing table, and placed on it the stomach, which was still entire, with the exception of two small cuts which must have been made in taking it from the body; witness slit it open with the scissors, and found a great portion of it thickly covered with a yellow pasty matter, looking like wet clay; all the apparatus used by witness was either entirely new, or had been carefully cleaned by him previously. As he was strongly inclined to think this powder was sulphuret of arsenic, he proceeded to treat it as such; he separated a small portion of the yellow substance with the spatula, absorbed the moisture on blotting paper, and then dried it; witness mixed it with a certain proportion of carbonate of soda and charcoal, both well dried, and put the whole into a reducing tube, and immediately sublimed metallic arsenic; this metallic arsenic was made the subject of the other experiments: witness first heated it, allowing the air to enter the tube, and it became oxidized, and sublimed into a white crust; this formed the second test of the presence of arsenic. Witness then introduced two drops of water into the tube containing the white crust, and with heat dissolved it; these two drops were made the subjects of two experiments. Into one he put a minute portion of ammoniacal sulphate of copper, and immediately found the green precipitate of Scheele, or arsenite of copper; into the other drop he put a minute portion of the ammoniacal nitrate of silver, and the yellow precipitate of the arsenite of silver fell. He had thus four tests of the presence of arsenic; it only remained to be brought back to its original state by passing a stream of sulphuretted hydrogen through the arsenious acid; this he did, and obtained the beautiful yellow of the sulphuret of arsenic; he had gone through these experiments four different times, and all with the same results. He next attempted to discover what quantity of the yellow substance there was left in the stomach; there was nothing of any importance in the stomach besides; he inverted the stomach into water, and washed its internal surface; he allowed the yellow powder to subside, and filtered the fluid so as to separate the yellow matter; in doing so he found that certain portions adhered to the internal surface of the stomach, and in two spots an infiltration had taken place into the substance of the stomach itself, the quantity collected could not be well separated from the filtering paper, on account of the animal matter; the nearest estimate by weighing, and counterpoising an equal sized piece of paper, gave seventeen grains as the weight of the substance and animal matter. To get rid of the animal matter he introduced (taking away four grains for the purpose of other experiments) the paper and its contents into a flask with nitric and muriatic acids, and boiled it till every thing was either decomposed or dissolved, except the fibres of the paper; and next filtered and extracted the whole of the fluid, and then precipitated the arsenic by means of sulphuretted hydrogen; the thirteen grains yielded witness four grains of sulphuret of arsenic. There are but two metals that will give the yellow precipitate with sulphuretted hydrogen; the one is cadmium, which is exceedingly rare, scarcely ever to be met with in this country; he had about half an ounce of it, which he should think is more than there is in all the kingdom besides; the other is the peroxide of tin, which is also scarce, and is not used at all as an article of commerce or medicine, and in this case could not possibly exist.—(Mr. H. then shewed to the jury the matter which had been taken from the stomach, and which had produced the yellow precipitate, or sulphuret of arsenic; and also specimens, in sealed tubes, of the action of all the other tests. The stomach itself was also exhibited, and the yellow spots were very apparent, and some of the yellow matter still adhered to its coats.)

By the Coroner.—He believed there is no well-authenticated case of how much sulphuret of arsenic will destroy life; the quantity will differ with the proportion of the materials used in manufacture; native orpiment is not so poisonous as the factitious; it is possible that it might have been swallowed in the form of white oxide, or arsenious acid; arsenic is in common called 'white' or 'yellow' arsenic, and would be sold by druggists under those names; white arsenic is chemically called arsenious acid, and yellow arsenic, sesquisulphuret of arsenic; they are both poisonous, but not equally so; white is the most poisonous; all compounds of arsenic are poisonous; the operation of each is nearly similar. He had never seen a stomach operated upon by arsenic before; should think the yellow spots were caused by the rapid operation of the arsenic suspended abruptly by death; if they had taken place after death, they would, in his opinion, have been as generally extended as the sulphuret itself; both sorts of arsenic are very cheap; he had not the slightest doubt of the nature of the poison found in the stomach; he could stake his existence on the fact of the presence of arsenic in the stomach and intestinal canal; cannot say in what state the arsenic was taken; the process of decomposition sometimes produces sulphuretted hydrogen, which would convert white arsenic into orpiment; that found in the stomach is orpiment; cannot positively say in this case whether sulphuretted hydrogen had been produced, because adipocire was formed, and the decomposition of the body did not proceed as is usual; he was certain arsenic could not have been introduced after death, as the stomach was whole when it came into his possession; witness's attention was first arrested by seeing a small drop of yellow matter ooze from the stomach; the great quantity of yellow substance in the stomach struck him at once that he should find a mineral poison. He observed no bile in the stomach; there was nothing of importance in the stomach; if there had been bile he must have seen it; he was certain there was no bile.

Cross-examined by Mr. PAYNE.—Sulphuretted hydrogen might enter into the stomach from other parts of the body, although there was no orifice; he thinks the yellow spots were produced during life; he had not seen many stomachs that had received poison; he had frequently the contents of stomachs sent to him to analyse; in the stomachs he had analysed he had never met with any arsenic; this is the first, and he hoped it would be the last; this matter in the paper and the tube are both from the stomach; it is the same as is sold in the shops for yellow arsenic; should think that the four grains he reserved, and what adhered to the coats of the stomach, with what he worked off, would yield 5½ or 6 grains of the pure sulphuret of arsenic from the stomach alone.

Mr. KELSON was then called, but stated that he was not aware that he could add any thing to Dr. Riley's evidence; it was so clear and ample.

Dr. J. A. SYMONDS, lecturer on Forensic Medicine at the Bristol Medical School, was present at the disinterment of the body of the deceased, and at the subsequent operations. His conviction was, that poison (sulphuret of arsenic) was in the stomach and intestines; he had never seen the body of a person who had died from arsenic before; the effects of an over-dose of arsenic would be extreme purging. Hahnemann, a German physician, states that two grains of white oxide of arsenic will destroy the life of an adult: the smallest quantity, in an actual well-authenticated case, is four grains; it is recorded by Zittman. Professor Christison, the first authority in this country, says four and a half grains have killed, and mentions the case. M. Guibourt, a French chemist, states the analysis of artificial orpiment to consist of 94 parts of arsenious acid and 6 of sulphur; this analysis is confirmed by Orfila; ordinarily a poisonous dose would operate in half or three-quarters of an hour; it varies with circumstances—it may operate much earlier."

Our readers cannot fail to admire the precision with which the main fact of the case—the administration of poison—was here ascertained. Thirty or forty years ago, medical jurisprudence was at so low an ebb in this country, that even the attempt to discover poison, after fifteen months' inhumation, would hardly have been attempted. Too much praise cannot be awarded to the medical officers—and especially to Mr. Herapath—for the talent which they displayed on this important occasion. We regret, with our contemporary of the Gazette, that a more exact description of the external appearances of the corpse is not given, after so long a sojourn in the grave. We are so little accustomed to such exhumations, that the question of identity might very much depend on such a description in future cases. We have no doubt that some of the medical gentlemen employed on this occasion, will furnish these particulars from their notes, or from memory, for the benefit of the profession.

---

### SIGNS OF PREGNANCY.

Under this head, Dr. Ingleby has published an instructive article in the December No. of our Dublin contemporary. His object is not to treat of the signs of pregnancy in general; "but to comment briefly, both on some of the supposed evidences of gestation, and the difficulty of forming a clear diagnosis—and also to advert to the signs which denote cessation of life in the embryo or fœtus, pointing out also their absence, modification, uncertainty and indeterminate nature." The following is the classification which Dr. I. adopts.

"I. The effect of vomiting.—II. Sanguineous discharges. — III. The state of the breasts.—IV. Twin cases, and the expulsion of foreign bodies from the uterus.—V. Fætor of the discharges.—VI. Ascent of the uterus, and the state of the cervix uteri.—VII. State of the hypogastrium, and examination *per vaginam.*—VIII. Obliquity of the uterus.—IX. Size of the abdomen apparently stationary.—X. Dropsy of the abdomen.—XI. Diminution of the size in the abdomen.—XII. Irregularity in the form of the abdomen.—XIII. Fœtal movements.—XIV. Auscultation.—XV. State of the funis.—XVI. Looseness of the cranial bones, puffiness of the scalp, desquamation of the cuticle, changes in the fœtal presentation, and sundry constitutional symptoms."

1. *Vomiting.*—This symptom usually ceases soon after quickening, though it sometimes continues to harrass the individual till the period of parturition. "These acts of vomiting are strongly in favour of the preservation of fœtal existence, and I do not recollect an instance of their continuing after vitality in the ovum had ceased. They are sometimes suddenly arrested by hæmorrhage. Venesection is one of the most powerful correctors of obstinate and protracted vomiting.

2. *Sanguineous Discharges.*—A single attack of hæmorrhage may prove fatal to the embryo, and even to the mother —and that, too, in the early stage of pregnancy. The fœtus may perish, and yet remain in utero till the ninth month. In some cases, the hæmorrhage will be almost unceasing for months, without injury to either mother or child. A discharge resembling healthy menses occasionally continues during the first two or three months of pregnancy, without apparently influencing gestation. Menstruation appears to be necessary to impregnation, though some women (though rarely) conceive while suckling. Amenorrhœa, when preceded by a bad state of health, is a very equivocal sign of pregnancy.

3. *State of the Breasts.*—The enlarged mamma becomes uneasy, with blue veins and prominent nipple; whilst the areola, instead of presenting the rosy circle around the nipple, acquires a yellow, or rather a dark colour, with increased diameter, and follicles yielding a moisture. This is occasioned by pregnancy *only*, according to Dr. Montgomery. It is a tolerably correct criterion for a *first* pregnancy; but not so certain for subsequent ones. The secretion of milk is not an infallible test of pregnancy, as the following case will shew.

"A young woman of spare habit of body, declared that she had attained the tenth month of her pregnancy; the usual morning sickness was followed by a discharge of milky serum, which kept her linen constantly wet, and subsequently, by movements which distinctly simulated those of the fœtus, and such as she had experienced in her two preceding pregnancies. On examination *(per vaginam)*, in place of an impregnated uterus, I detected a firm tumor, the size of a large egg, situated at the posterior and lateral parts of the neck and body of the uterus, and bulging into the rectum; menstruation was regular, but, as might be expected, excessive. The pulsations of the abdominal aorta were very strong, but she declared that these were not the movements which occasioned the deception. I could not determine their character."

Enlarged mammæ, from disease, usually disappear after a few weeks, though the disease itself may go on progressing. The following are the changes, according to our author, which commonly result from extinction of life in the ovum.

"The areola loses a portion of its darkness, the follicles and nipples shrink, the nipples cease to yield a serous or milky discharge, and the mammæ suddenly diminish in bulk. Towards the close of an ordinary menstrual period, the congestion of the mammæ, which immediately precedes the act, begins to subside."

Passing over the section on twin-cases, which we cannot abridge, we come to—

4. *Fœtor of the Discharges.*—This is regarded as a prominent sign of extinction of fœtal life. "Blood, when confined only for a short time within the uterus, and exposed to atmospheric influence, soon becomes fetid." *Query*, How does atmospheric air get at blood confined within the uterus? We believe there is a medico-legal question now pending—perhaps decided—respecting the putrefaction, or supposed putrefaction, of the dead fœtus in utero. Does the fœtus become decomposed in utero, when deprived of life?

A high degree of fœtor may take place in the natural mucous secretion which lubricates the vagina—a peculiarity compatible with a healthy constitution and a living child. "A very fetid state of the liquor amnii is quite compatible with vitality in the ovum."

5. *Ascent of the Uterus—State of Os and Cervix Uteri.*—Although quickening takes place about the fourth month, the ascent of the uterus is very uncertain, being regulated partly by its own development—partly by the size of the pelvis. It will ascend prematurely when the liquor amnii is in excess, or when the cavity of the uterus is distended by fluid, the product of disease.

"In the practice of my friend, Mr. George Elkington, a woman was seized, in the early weeks of pregnancy, with an active hæmorrhage from the uterus, and it was supposed she would miscarry: presently, however, the case was rendered unusually obscure, by the vastly disproportionate increase of the abdomen. Instead of the uterus being found near the brim of the pelvis from the third to the fourth month, the abdomen had become both suddenly and generally distended by it. Vigorous contraction soon came on, and the expulsion of an immense quantity of hydatids, and a small fœtus was the result. Hydatids simulating natural pregnancy is not an unusual circumstance."

Our author is not aware that the presence of hydatids can be distinguished from a healthy state of the ovum. They produce a train of symptoms characteristic of early conception—and their evacuation is generally attended by a train of symptoms resembling abortion. Our author relates a case, where the premature elevation of the uterus appeared to be influenced by "an unusually large pelvis." On examination, before she could have passed the *third* month of utero-gestation, he found the body of the uterus enlarged, and the summit of the fundus within an inch of the umbilicus. Expulsive pain followed, and terminated in the discharge of the liquor amnii, and a very small fœtus and placenta, apparently of about eleven or twelve weeks' growth. For the life of us, we cannot see how the foregoing case proves "an unusually *large* pelvis." To our apprehension, it would seem to prove just the reverse—unless plenitude of capacity means, on the other side of the Channel, diminution of size. "From the fourth to the sixth month, *ballottement* of the uterus will afford very decisive information; but although we thus obtain an assurance that its cavity contains a child, we may remain in ignorance whether or not the child possesses vitality." Our readers will perceive that, a little before, Dr. Ingleby tells us that hydatids cannot be distinguished from natural pregnancy—how, then, can the "*ballottement*" afford us "decisive information"? Those of our readers who are not acquainted with French terms, must not suppose that "*ballottement*" means "balloting"—though it is curious enough that there is some similarity in the two operations.

The gravid uterus may remain within the pelvis as late as the sixth month, of which an example is given.

"Mrs. T. ætat. 20, has been married about a year, and the last act of menstruation terminated in the first week in January, 1834. The non-recurrence of the catamenia was not followed by any of the early signs of pregnancy, but about the end of May, a peculiar fluttering sensation favoured the idea of pregnancy. The fluttering continued very sensibly increasing until the end of June, when it entirely ceased. Accustomed to menstruate with great regularity, no doubts were entertained respecting the existence of pregnancy; until it was discovered that the abdomen had not at all increased in size. I was consequently desired to see her on the 10th of August.

There was neither vomiting, sickness, discharge, sense of weight, coldness nor pain; the breasts were slightly enlarged, the superficial veins peculiarly distinct, and the areola but imperfectly formed; the abdomen was in no respect enlarged. Notwithstanding the patient's spare habit, I could not feel any part of the uterus above the brim. On internal examination, I found the cavity of the pelvis occupied by a large tumor, resembling the head of a child, which had descended within nearly an inch of the vaginal orifice. The cervix uteri had very nearly disappeared, and the os internum was soft and slightly open. Although the *ballottement* of the uterus was quite impracticable, I felt certain of the fact of pregnancy, and testified accordingly, but my opinion as to the vitality of the ovum was very guarded. Four days after this visit a slight hæmorrhage ensued, which was followed by pains, and the expulsion of a still-born and decomposed six months' child. The gravid uterus, instead of rising out of the pelvis at the fourth month, may lie completely beyond the os externum. In one instance of this kind, I succeeded both in returning the uterus within the pelvis, and retaining it there by means of a very large globular pessary, until the natural elevation had taken place."

The os uteri acquires a laxity of texture soon after conception, which it does not present at other times—but the cervix uteri undergoes little change, antecedent to the fifth month. About this time, the development of the structures commences; "but this development may be occasioned by an extraneous tumor, in degree almost as great as by the growth of the ovum."

6. *State of the Hypogastrium—Examination per vaginam.*—We shall extract the following passage, as it cannot be abbreviated.

"Inaccuracy in diagnosis is generally the result of our examination being made in a desultory manner, and in unfavourable positions of the body. For the purpose of examining the uterus above the pubis (the bladder and rectum being previously evacuated), the body should be supine, the head and shoulders being rather elevated, and the abdominal muscles relaxed. The hypogastric and iliac regions must be carefully explored, and if a hard body be felt, the fingers should be applied so as to ascertain, if possible, its volume, form, consistency, mobility, and connexion with other organs.* Examination *per vaginam* may be conducted in the same position of the body, but it is sometimes advantageous to make this examination when the patient is erect, by which the size and weight of the uterus, if not its amount of elevation, will be more correctly determined. It may be advantageous to conduct the super-pubic examination in the same posture, making the requisite allowance for the comparatively relaxed state of the abdominal parietes in women having previously borne children. Percussion of the abdomen, when properly performed, is strikingly advantageous in determining the nature of abdominal enlargements, whether occasioned by a solid body, the evolution of gas, an excess of liquor amnii, or other fluid depositions. In pregnancy the fundus uteri will not be felt above the pubes until the end of the third month, and there is no *visible* increase of the abdomen before this period, but afterwards the enlargement will be progressive. When the centre of the hypogastrium is rendered prominent, and even (varying a little in these respects with the motions of the fœtus) moderately firm, or slightly elastic, the outline being defined, and having the intestines on either side and above the tumor, such condition presents the strongest characters of pregnancy.

The contrast between the state of the hypogastric and epigastric region, from the fifth to the seventh month, especially when the patient stands erect, is very marked. There can be no difficulty in ascertaining whether the uterus *is* or is *not* enlarged, but whether the enlargement is occasioned by conception, may be less easily determined. Great stress has been laid upon the state

---

* Boivin and Dugès, translated by Heming, page 31.

of the umbilicus, which in early pregnancy may be actually more retracted than natural, on account of the uterus being more or less prolapsed. In advanced pregnancy, the umbilicus will either be on a level with the surrounding parts, or project beyond them. The appearance of the abdomen, notwithstanding the elevation of the umbilicus, may greatly deceive us. It would appear improbable that a distended state of the abdomen, from visceral enlargement, should be confounded with the gravid uterus; but it must be recollected, that the shape of the abdomen may in no respect differ from a state of advanced pregnancy, and the patient may also experience the constitutional evidences of that state of the system."

Some curious cases are given of mistakes respecting pregnancy, and where substances in or near the uterus gave rise to suspicions of utero-gestation. We must refer to the work itself for their details.

7. *Obliquity of the Uterus.*—This is referred to three causes—1, to deformity of the pelvis and spinal column—2, to distended states of the colon, the ordinary form of obliquity described by authors—3, to relaxation of the abdominal coverings. Obliquity is rarely noticed in first pregnancies, on account of the resisting state of the abdomen; and the third form is rarely seen before the seventh or eighth month, when its nature cannot be misunderstood. When it occurs about the fourth month, its cause and nature are often obscure.

8. *Stationary Size.*—The abdomen may acquire an ordinary and proper degree of enlargement at a certain period of utero-gestation, and afterwards cease to alter its dimensions, though the fœtus be alive.

"A woman, four months pregnant, and who had quickened about a week, was suddenly seized during the night with a copious discharge of the liquor amnii, which recurred frequently in drainings and occasional gushings, attended with pain, and followed, at times, by hæmorrhage. These discharges continued without intermission till within a few days of her delivery, which occurred shortly after the seventh month. During the three intervening months the abdomen did not visibly enlarge, and although the movements of the fœtus were not felt after the first appearance of discharge, it was born living, but very feeble. From the sudden arrest of size, the friends of the woman were incredulous as to the existence of pregnancy. Under these and similar circumstances, as already observed, the neck of the uterus will not shorten, or more correctly speaking, not develop its structures, until an unusually late period of gestation."

9. *Dropsy.*—Pregnancy and ovarian dropsy often occur simultaneously—the general health being frequently but little affected by the latter disease. But the excitement of pregnancy may give the ovarian complaint a malignant tendency. On the other hand, all dropsical tendencies are checked by delivery. Tapping, during pregnancy, should be avoided, if possible, on account of the rapid reproduction of the fluid, and the tendency to peritoneal inflammation. Auscultation seldom affords us conclusive information in the way of diagnosis.

10. *Diminution of Size in the Abdomen.*—This change is peculiarly striking, when the fœtal life becomes extinct, although the ovum may be retained in utero until the full term is expired. "A woman expressed her conviction that she was in the third month of pregnancy. Soon after this time, although she lost every symptom of pregnancy, amenorrhœa excepted, she persisted in asserting its existence. At the ninth month labour came on, which terminated in the expulsion of an apparently three months' fœtus (not decomposed) and a diseased placenta. Not only, however, does the abdomen diminish in bulk when the fœtus is deprived of life, and the blood is in a great measure diverted from the uterus, but also when the vitality of the ovum is unimpaired, and the vessels of the uterus are undergoing a progressive increase."

11. *Fœtal Movements.*—These are the only incontestable evidences of the life of the fœtus—yet these movements have deceived the most experienced practitioners. "When the hand is placed over the gravid uterus, the sensation imparted to it by the movements of a living child varies from mere knuckle-like substances, felt weakly, or passing slowly from one portion of the uterus to another, to that peculiar ictus, or strong and sudden jerk, which is so characteristic of pregnancy."

In consequence of the light and distended state of the abdomen towards the close of pregnancy, no distinct sensation can be felt by the hand, when the body is erect; but when reposing on the back, the movements may be distinctly felt. It is proved by daily experience, that fœtal movements may be simulated by the action of muscles, the presence of hydatids, a slight ovarian enlargement, and other growths and morbid depositions, both solid and fluid—even bodies of air moving about in the intestines are frequently confounded with the movements of the child.

"An involuntary movement of the muscles of the abdomen may be occasioned by any large body within its cavity, irritating the muscular fibre. These movements vary from a mere twitching, to a strong retractation, especially about the umbilicus. I examined a woman having an enlargement of the abdomen, chiefly from fat, in whom these muscular retractions were so marked, that several experienced females would not abandon their conviction of the fact of pregnancy, although the supposed period of gestation had expired upwards of ten weeks. I take it for granted, that twitchings of the recti muscles will generally be more or less painful. An apparently creeping movement, simulating the motions of a feeble infant, and occasioned by large bodies of gas in the intestines, may be distinguished by the hand, notwithstanding the intervention of a considerable substance between the intestines and the abdominal integuments. On one occasion I was greatly misled by these movements. I was desired to see a woman in whom a tumor had become developed on the left of the umbilicus, and on a line with it. It was slightly movable, at times painful, but not on pressure; projecting considerably forward, sloping from its summit, and being about the size of the gravid uterus at the fifth month of pregnancy. Its texture appeared to be firmer in some parts than others, but this was owing to partial adhesions having formed between the general and uterine peritoneum. It afforded no sense of fluctuation, but on placing the hand over the surface of the tumor, a distinct crawling movement was traced in every part of it, but unaccompanied by the sudden impulse before alluded to. On internal examination, although the os uteri and the vaginal portion of the cervix were scarcely changed, the superior part of the cervix had degenerated into a tumor, quite as large as a child's head, exceeding it in firmness, but without the resilient property of an immature fœtal skull. Repercussion could not be produced. The tumor continued enlarging, the sufferings of the patient progressively increased, and the result was fatal. On examination, *post mortem*, it was found that the inferior part of the uterus had degenerated into a very thick and fibrous, or fibro-cartilaginous texture. The fundus and body of the organ were converted into a large sac, not thicker than an ox bladder, and contained three pints of dark, fœtid, muco-purulent fluid, of the consistence of gruel. The mucous and fibrous structures of these parts of the uterus had nearly disappeared, and the peritoneal coat, though generally thickened, had ulcerated in one spot, where, from its thinness, it burst during the examination, and allowed a part of the fluid to pass into the abdomen. The fluid was prevented during life from escaping into the vagina by a quantity of tenaceous mucus which lined the sides of the cervix uteri, as in ordinary pregnancy."*

* "The *post-mortem* examination was made by my friend, Mr. J. M. Coley, of Bridgnorth, who kindly furnished me with a copy of his notes."

The crawling motion alluded to was probably produced by strong peristaltic movements of the colon, felt through a fluid of moderate density—or, otherwise, by the thick fluid in the uterine cavity changing its position when the hand was placed over its attenuated surface. In a few cases, the movements of a living child have not been felt at any period, either of pregnancy or labour. This may depend on an excess of liquor amnii, torpor of the uterus, excessive uterine action, or feebleness of the infant, especially after hæmorrhage. The movements of an infant may be almost paralyzed by the full and unremitting action of the ergot of rye. Our author is very unwilling to administer this remedy, lest a dead child should be the consequence.

"Another source of deception consists in confounding inanimate with animate movements; an accidental change in the situation of a dead fœtus, for example: a woman who was most anxious for a living child, persisted that she felt the child move at the beginning of her labour, and yet the cuticle was entirely detached, and decomposition far advanced."

12. *Auscultation.*—Without any pretension to be an expert auscultator, our author has distinctly detected both the soufflet and the pulsation of the fœtal heart, by the stethoscope and the naked ear. "But the soufflet is common to several diseases, and it is, therefore, an uncertain evidence of pregnancy. The sound will be rendered obscure, when the centre of the placenta corresponds with the centre of the fundus uteri on its posterior surface, and will be scarcely perceptible when the placenta is attached to the uterine orifice." The pulsation of the fœtal heart is, of course, conclusive of the presence of a living child. It is not distinctly heard before the fifth or sixth month—at least our author has not been able to distinguish it before that period. Some concise observations are made on the state of the funis, and on that of the cranial bones, &c. which we need not examine. The paper contains many useful hints and practical remarks, which we have transferred to our pages in an abbreviated form, as beneficial to our readers, and the best compliment we could pay to the author.

---

### THE GUMS, THEIR DISEASES AND SYMPATHIES. By Mr. WAITE.

We have heard that Mr. Waite is a scientific dentist, and we have no doubt that he has studied the subject on which he now writes, not only with care, but with the advantage of a general and proper knowledge of medicine in its full extent. We cannot help suspecting, however, that, like a physician who has lately published on the same subject, Mr. W. has estimated too largely the influence of affections of the gums on affections of other and distant organs. This error seems almost inseparable from exclusive attention to a single subject in practice, however extended may be the previous study. Mr. W. exhibits classical learning as well as pathological research in this little volume; but we think he is not a practised writer, many of his sentences being obscure or involved. "To the medical world (says he) with all respect to their great exertions and intents, I would submit the teeth as organs most essentially conducive through life to a healthy temperament and digestion." Here we are left in doubt whether Mr. Waite recommends teeth to the doctors, or the study of them. We must pass over a great many sections on the structure and functions of the teeth and gums. At page 59, we have a chapter entitled "the influence of the passions of the mind on the gums." The far greater part of the chapter is occupied with quotations from the poets and others, illustrating the general effects of the exciting and depressing passions on health—such as the story of Sophocles dying as soon as he was proclaimed victor in a dramatic contest, &c. There are only a few lines in this chapter on the gums themselves as participating in the state of the general health. There are other chapters, however, in which Mr. W.

keeps more closely to his subject, and offers many useful observations, which we are unable to condense or collect, owing to the loose manner in which the Essay is written. We shall merely offer an extract, as a sample of the matter.

"A change of residence to a damp climate will often rouse up in the gums a great degree of vascularity. In the damp places of England and Ireland, the appearances which the gums present are of a turgid and vascular nature. In the damp countries of France these conditions of the gums run a much greater length from the circumstance of the difference in the constitutions of the two nations. In the damps of Germany and Switzerland persons also lose their teeth early in life, the climate engenders malaria and low fever, enfeebles the powers of digestion, and brings on rheumatic affections with languor and general constitutional debility.

A morbid vascularity of the gums is oftentimes produced by cerebral affections, by close scholastic application, by a state of fear and anxiety in which youths are kept, added to the manner in which they are often made to pore over books by parents who themselves are untaught in the real doctrines of nature.

We have already enlarged on the sympathy between the stomach and the gums, an altered appearance of the one coinciding with the same phenomena which may be distinctly traced to the other; hence dissipation and vice in young persons produce derangement of the stomach; transmitting its characteristics to the gum. The young female, emerging from the nursery to the nocturnal scenes of London revelry, is also subjected to disease of these organs, the gums as well as the stomach sympathizing with the state of excitement she is then kept in. Here we may also bring in the man of literature and close application; the tradesman, exposed to hot rooms, and capable of enjoying but little air and exercise; the statesman, borne down by the trammels of office, with its anxieties and perplexities; the unhappy hypochondriac; and, lastly, the maniac himself.

The period of pregnancy often produces a diseased alteration in the gums, which occasionally loosens and destroys the teeth; this comes on from the unsettled state of the circulation during that important period.

Thus far I have endeavoured to explain some leading facts connected with the diseased appearances of the gums. There remains still another important pathological sympathy which must not be passed over. I allude to a morbid state of sensibility in which they are frequently found; this state is roused in the gums to a considerable extent by the effects of scarlet and other fevers when severe, by the after effects of mercury when given in an oriental country, as also by all circumstances which tend to disease the general state of the nervous system.

I lately witnessed a remarkable case: —A young gentleman had just returned from India, where he had taken mercury to a very considerable extent. He had fallen into a regular state of hypochondriasis; he had sensations every now and then over him of momentary dissolution occurring, and of these he evinced the greatest horror; he dreaded the appearance of a razor, of a knife, or of any instrument which could inflict a wound; his gums had receded from the teeth; their edges were thickened, but of a firm consistency; these were very painful to the touch, as also were the necks of all the teeth where the bony part was exposed. I used my utmost endeavours to persuade him that he would soon be well, and under medical guidance he went into the country. In cases of tic doloureux, especially in elderly females, the gums frequently possess extreme sensibility. I recollect a lady who was dreadfully afflicted with tic doloureux. Her gums were soft, and it appeared as if most of the capillary vessels in them had sloughed, and that a lymph of a light brown pellucid colour, coinciding with the state she was in, was underneath their surfaces.

Sensibility is also often roused in the gums to a great extent by heat of stomach and cold: it also succeeds the vascular condition, roused by the many circumstances we have considered, which bring on an inordinate circulation through them."

The work will prove useful to the general reader, and is not undeserving of professional perusal.

---

German Lunatic Asylum.

"The insulated rock, on which stands the Irrenaustalt, rises abruptly from the plain, and commands a rich and romantic view, bounded towards the south by the peaks of the *sieben gebirge*,—towards the north by undulations over which the towers of Cologne are just discernible,—on the east by a chain of low wooded hills,—whilst, towards the west, the eye is attracted to the wide expanse of Rhine, which flows so majestically amidst gardens and vineyards, spired villages, and ruined castles. At the foot of the rock stands the old town of Sieburg, whose crumbling ramparts are bathed by the Sieg, a mountain torrent that, after meandering a couple of leagues, precipitates itself into the Rhine. In the eleventh century, the mountain, or rather craggy hill, of Sieburg was crowned by a castle belonging to the Count Palatine Henry, who presented it to the Archbishop Annon. The latter established there a Benedictine monastery, erecting for this purpose a vast and stately edifice. Napoleon who everywhere appropriated monastic properties to the purposes of the state, expelled the humble Benedictines from their splendid residence: and after the peace, the Prussian government having failed in finding a purchaser, the building was, by a few additions and alterations, converted into an Irrenaustalt, or asylum for the insane of the Rhenish provinces. Nothing can be better adapted to such an institution than the long corridors and separate cells which form the interior of a monastery. The edifice is nearly quadrangular, but its great central court is divided by a noble church, which towers above the rest of the structure. On three sides the ground-floor is almost entirely occupied by the kitchen, baths, and offices: the cells of the first floor are principally devoted to the poor patients, whilst those of the second are inhabited by the *pensionnaires*, or persons of a higher class, who are admitted on terms proportioned to the accommodation which they require. The fourth side of the quadrangle, which offers the advantage of being a little separated from the others, has for its inmates the more restless or noisy patients of all classes, the ground-floor being assigned to the men, and that above to the women. At present there are about two hundred patients, of which only eighty are females.

There is one peculiarity in the moral treatment adopted in this institution, which is especially worthy of notice—the employment of labours, either mental or bodily, as a remedial measure. With this view, a distinction is made as to the habits and relative education of the patients. Of the poorer inmates, the males are, with but few exceptions, employed six hours every day in the cultivation of the gardens and fields which surround the hill; whilst the females either spin or are actively engaged in the domestic arrangements. In their leisure hours, those who are recovering, meet in rooms set apart for society, in which mechanical games, the journals, and works of a light and instructive character are introduced for their amusement. The *pensionnaires*, in general persons of good education, are also called into activity—the ladies to exercise themselves in needlework, reading, or music—the gentlemen to pursue literary or arithmetical studies proportioned to their abilities, and adapted to the peculiarities, of their cases. The occupations of both are carefully superintended by well-informed persons of both sexes, the literary exercises being more particularly revised and corrected by the Protestant and Catholic clergymen of the establishment. One gentleman, whom I visited, was translating Cæsar's Commentaries; a second was learning a new piece of music; and a third, observing that I was a foreigner, conversed with me in Latin. The private apartments of the *pensionnaires* are supplied with every necessary comfort, and the courts and gardens afford ample space for recreation and exercise. There is a library, and in the public rooms, billiards, and various kinds of games and musical instruments serve to while away the few hours which are not expressly devoted either to bodily exercise or to study. Idleness is banished, and with it much of the melancholy which is so usually observable among the insane: order, neatness, and industry, reign in every branch of this interesting establishment, the arrangements of which reflect infinite credit on its learned and scientific director, Dr. Jacobi."*

---

MEDICAL PROBLEMS. By DR. GRIFFIN. ENTERITIS.

In our Dublin contemporary for January last, Dr. Griffin, whose work we fully reviewed in our last number, has published a lengthy paper on the nature and treatment of enteritis, which we deem worthy of notice in our Periscope. The gist of the inquiry bears on the propriety or impropriety of purgatives in enteritis. The evidences of various authors, from Parr to Armstrong and Abercrombie, are adduced, compared, and contrasted. A case is then detailed, which is as follows, though much condensed.

A lady, aged 32, was attacked in the evening of November the 1st, with pain in the lower part of the back, and weariness, which three glasses of Port wine did not relieve. She was feverish in the night, and next morning had a dose of castor oil. At 3 P.M. she had severe pain in the stomach, and the whole of the abdomen was found to be tender, and somewhat tumid. She was hot, restless, and exhibited a painful expression of countenance. The castor oil had operated freely three times. She was placed upright,—a vein opened, and eight ounces of blood abstracted, when she fainted. She was then placed in the horizontal position, had some warm drink, and again bled to two ounces, when syncope supervened. Twenty-four leeches were then applied to the abdomen, especially to the parts most exquisitely tender. A dose of calomel and opium (3 grs. of one, and a half of the other) was given, and a dose (2 grains to one) to be given every second hour afterwards. In the morning of November 3, the pain still continued very severe, and the tenderness exquisite. Twenty ounces of blood were taken from the arm, without faintness. The bowels being unmoved since after the castor oil, and the abdomen somewhat tumid, pills of aloes and hyosciamus were exhibited, and a lavement thrown up, containing some oil of turpentine.

"On administering this last she was seized with a dreadful forcing or bearing down pain in the anus, and passed nothing; the pain seemed as excruciating as any that could occur in violent labour; lasted for about twenty minutes, and was then relieved by the warm bath. In two hours afterwards a simple injection of oatmeal tea was given, followed by similar suffering, and was in like manner retained. The permanent pain was at this period severest in the left iliac region and about the navel, where the tenderness on pressure was extreme; the countenance was more anxious; the tumidity of the abdomen was increasing, and the stomach beginning to reject the drink. In consultation with Dr. Geary, Sen. to whose judgment on these occasions I have been often indebted, it was now agreed to take blood again, and eighteen ounces more were drawn, being the third general bleeding within twenty-four hours. Two grains of opium and a grain of calomel were given immediately after, and ordered to be repeated every two hours through the night."

There was considerable amendment next morning, the 4th November. The pain and sickness had subsided—the injections had come away, with thin feculent matter. Still there were some unpleasant symptoms remaining, and

---

two grains of opium, without calomel, were given, every two hours. In the evening, the symptoms were still more favourable, and she spent the night, and the following day (5th Nov.) with little uneasiness—but she mentioned at one time, that fæces were passing from the rectum. This circumstance was not attended to, and the opium was ordered every fourth hour. She had taken 32 grains within the preceding 32 hours, without stupor or head-ache.

"On the next evening, as she lay on the sofa while her bed was making, she felt a solid substance passing from the rectum, which alarmed her terribly. On examining, I found a rope of sloughy stuff, soft and purulent outside, but tough and fibrous within, not unlike the ischiatic nerve in a decayed state, hanging from the anus for the length of a foot or more. On attempting to draw it away, it appeared to be still adherent within the gut, and she complained of pain. After a little, however, it was removed without much effort, and a gush of matter to the amount of perhaps two table spoonfuls followed. The slough was about the thickness of the thumb, or more, and was fifteen or sixteen inches in length. We at first supposed it was a portion of the small intestine which had mortified, and been thrown off; but on close examination no distinct traces of a canal could be found. Sometime after an injection of warm water and sweet oil was administered, which came away in about twenty minutes mixed with some matter, but without any appearance of fæces. I now introduced the finger into the rectum, and felt at the posterior part, close to the sacrum, a rugged irregular edge, as if it was the termination of the part from which the slough had been cast off: the examination gave much pain, especially when I pressed within upon the intestine. Several days passed without much alteration in the case, there was matter daily discharged to the amount of three or four ounces, and there was at times severe dysury, at last demanding the use of the catheter. The urethra was blocked up with a thick mucous discharge, which closed the common instruments, and prevented the waters escaping until a very large-sized flexible one was introduced. Sometimes the difficulty appeared to be connected with mere nervous irritation, as she occasionally got sudden and unaccountable relief, the water coming off without any very obvious reason. Three days had now elapsed since the subsidence of the pain and tenderness of abdomen, and six days since the bowels had been moved. There appeared to be some fulness of the abdomen, and she began to feel uneasiness again in the left iliac region; we had allowed the bowels to remain so long at rest with a view to the healing of the ulceration in the rectum, and from an apprehension, that much disturbance of the intestine might increase the inflammation and sloughing."

A dose of castor-oil was given, followed by pills of aloes and henbane, which operated freely in the course of the day (Nov. 8th), the motions being thin, dark, and streaked with matter. The pain in the left iliac region became alarmingly increased, and extended rapidly over the whole abdomen, with a return of the restlessness and distress of countenance, sickness, &c. The pulse became feeble and rapid—thirst extreme—belly tense—countenance sunk—face covered with cold perspiration. In this distressing dilemma, it was determined to trust to opiates, as general depletion was out of the question, and purgatives might increase the evil. Three grains of opium were given for the first dose, and two every two hours afterwards. Twelve leeches were applied to the abdomen, with fomentations, &c. "The effect was wonderful; the pain and tenderness gradually subsided—the vomiting ceased—the pulse became slower—and she got some sleep." The amendment went on for the ensuing five or six days—excepting some dysury, and the continual draining of matter from the rectum, with pain and soreness inside the sacrum. After the 9th, the opium was gradually diminished. The appetite returned, and the tongue became clean. After seven days without an evacuation, the left iliac region became painful, with feverishness, and disposition to vomiting. An opiate was first given, and followed by calomel, with mild doses of castor-oil and jalap, until the bowels were freely moved. Great relief ensued, and the abdominal tenderness finally subsided. Still there was much to be apprehended, after a slough of fifteen or sixteen inches had been detached. A mild purgative was given every fourth day, while opiates were given in the intermediate periods. At the end of three months she could move about the room, and, at the termination of the fourth month, she was perfectly recovered.

It will be evident to our readers, that the foregoing case will not afford many data for grounding principles of general treatment, in acute and simple enteritis. Sloughing of the rectum, and most probably of the sigmoid flexure of the colon, is not an every-day occurrence. Dr. Griffin, however, thinks that the case in point bears strongly on the general principles of treatment, in respect to some particular items—as purgation and mercury. As to blood-letting, we shall be brief. We agree with our author, that the precept of placing the patient in the upright posture, in order to induce early syncope, is more than doubtful. We conceive it to be erroneous and dangerous. It is not mere syncope which arrests an inflammation of the intestines or other internal organ—it is the detraction of blood from the whole system, as well as from the part inflamed, so as to withdraw, as it were, the basis on which the phlogosis is founded. Before that condition is attained, syncope only interrupts, instead of accelerating, the means of cure. It is pretty evident that it did so in the foregoing case, and we have often seen the same effects produced by the same means.

The chief point of discussion hinges on purgation in enteritis. Dr. G. admits it as an undeniable fact, that—"in a great number of cases of enteritis, the salutary termination of the complaint has been by free evacuations from the bowels, and that, before this occurs, perfect relief is seldom obtained." But on the other hand, he observes that, as some cases occur in which the complaint goes on to a fatal termination, the bowels being free all the time—while others, again, as the case just detailed, admit of perfect relief, though the bowels are locked up for several days—we must pause—and, perhaps, come to the conclusion, that we are mistaking effects for causes—in other words, "that patients recover, not because they are purged—but they are purged because they recover." There is probably more point than propriety in this sentence. But we will not enter fully into a consideration of Dr. Griffin's advocacy of opium, and rejection of purgatives, after bleeding in enteritis. The plan was tried, in consequence of Dr. Armstrong's paper, and it did not answer fully the expectations of that talented, but somewhat enthusiastic physician. If depletion, general and local, has so far reduced the enteritis that irritation *only* remains, why then the opium alone will answer the object in view. But if any inflammation be left behind, the opium, by itself, and especially in such doses as are recommended, will mask the enemy, increase the constipation, and jeopardise the life of the patient. But if we subdue, as far as possible, the enteric inflammation, and then combine calomel with the opium, we are securing a safe evacuation of the bowels, while we are allaying their irritability. We advocate as little as Dr. Griffin does the employment of mere purgatives in the early stage of enteritis; because we know that they will generally fail in opening the bowels, and may do harm by increasing the peristaltic motion. But we do advocate the administration of calomel and opium immediately after free depletion, and, after a reasonable lapse of time, the exhibition of castor-oil or other mild purgative, aided by emollient lavements. We know, by a tolerably wide range of experience, that this is a safer practice than that of large doses of opium after bleeding. Dr. G. talks a great deal about the danger of "*stimulating* the inflamed parts, or the parts contiguous," by purgatives. What! does Dr. Griffin pretend that calomel, applied to the mucous membrane of the stomach and intestines, acts merely as a "*stimulant?*" Accurate observation would teach him that it acts more as a *sedative* than as a stimulant; and, by increasing all the glandular secretions, and removing the contents of the intestines, whether healthy or vitiated, it removes one of the worst of stimulants. We are surprised that Dr. Griffin, for whose professional acumen we entertain a high respect, as our readers are aware, should thus condescend to play upon words, and pervert their fair meaning.

"Is it absolutely true that we can *violently stimulate* a mucous surface which is healthy, in connexion with a muscular or serous that is inflamed, without increasing the inflammatory actions in the latter?"

This passage is applied to calomel in enteritis!! A *violent stimulus!* why he could not say more, if we exhibited bumper after bumper of whiskey to a man labouring under enteritis! On cool reflection, Dr. Griffin will see the impropriety of the terms which he has used on this occasion. It is vain to bring in perforations of the intestines, as exemplifying the good effects of opium—for, in such cases, we can do nothing but soothe the sufferings of the patient.

We would just hint to Dr. Griffin, in conclusion, that the case of the lady, above detailed, does not hold out the most flattering picture of the beneficial effects of the treatment recommended. For our own parts, when called to a case of enteritis, we should not like to compound for a slough of the rectum and colon, "the length of a foot or more," even with the assurance that opium and constipation of the bowels would ultimately preserve life. By this observation, we do not mean to find fault with Dr. Griffin's practice in the particular case, believing sincerely that it was highly judicious. We only mean to enter our protest against the general adoption of the plan recommended—and against the conclusions which Dr. G. has drawn from the case in question.

---

### INJURIOUS EFFECTS OF SALT. By W. MATEER, M.D.

[Dublin Journal.]

After adverting to the many unfounded and ridiculous notions entertained by our forefathers respecting the salubrity and insalubrity of culinary salt, as an article of diet, Dr. M. proceeds to give us the result of his own observations as an out-door visiting physician of the Belfast Dispensary.

"As not only the really poor, but also the working classes, apply for relief, such cases are very numerous. Those which fell under my notice presented a striking uniformity in their symptoms. Nearly one-half of the adults complained of the same kind of indisposition. The symptoms so generally complained of were great weakness, lassitude after any ordinary exertion, a feeling of soreness through the whole body, and a sensation at the region of the heart, which the patients themselves differently described, as a 'crushing,' 'tearing,' and 'gnawing' at the heart. There were also palpitations, stitches through the chest, with a catching cough, dyspnœa in a greater or less degree, and costiveness of the bowels. The appetite was, for the most part, unimpaired, which sufficiently distinguished this complaint from dyspepsia; neither was there present flatulency, or the burning and acidity of the stomach, which characterize this disease. The stitches in the chest, and short cough (when present) might readily have caused them to be mistaken for some affection of the chest, but the feeble pulse, the shifting of the pains, and the existence of other symptoms proved that they were merely sympathetic.

These complaints were found only among the lower classes, the higher being, as far as my observation has extended, quite exempt from them. This circumstance would naturally lead us to refer their origin to some deficiency in cleanliness, clothing, or diet. There are none of these circumstances which marks the difference in the conditions of society, so much as the nature of the diet. In the case of the poor, it consists in a great part of salted provisions, which are but sparingly used by the rich."

These facts induced our author to attribute the symptoms above-mentioned to the inordinate use of *salted provisions* ;—and we are inclined to agree with him in a great measure. We conceive that the above symptoms result from want of nutrition, in consequence of the indigestibility of the food thus hardened and preserved by means of salt. We are disposed to think that if flesh, fish, &c. could be preserved and rendered hard and difficult of digestion by any other material than salt, the same or nearly the same train of symptoms would be the result. This reasoning is not subverted by the statement that—" the entire disuse of salted provisions, and a diet of fresh vegetables and flesh meat, continued for some time, always afforded relief." It is well known to those who have served on foreign stations during the late war, that ships' crews coming into port affected with scurvy, were soon cured by plenty of fresh meat, *independent of vegetables*, and eaten with abundance of salt. In short they recovered much sooner from scurvy, when on exclusively a fresh meat diet, than on one exclusively vegetable. In the physiological and pathological reasonings of ~~our~~ author on this subject, we cannot entirely agree, inasmuch as he looks too much to the mere action of *salt* on the constitution, and too little to the deleterious effects of *salted*, and consequently *innutritious and indigestible food*. Nevertheless we return him thanks for his communication.

---

### Cases of Uterine Inflammation. By Fleetwood Churchill, M.D.

[Dublin Journal.]

Two cases are detailed in this short paper—one of them very succinctly.

*Case* 1. " Mrs. M'Quillan, æt. 45, lymphatic temperament, was taken in labour, May 20, 1834. The pains were tolerably effective during thirty hours, and then became irregular. The head of the child presented in the first position ; it descended into the pelvis, and there remained unaffected by the pains during the succeeding eight hours. As the difficulty appeared to arise from feeble and irregular uterine contractions, ergot of rye was ordered. She took three doses, each containing a scruple, at intervals of half an hour. At the time the first dose was given the pulse were ninety-six : in six or seven minutes they fell to sixty-nine, accompanied by a feeling of faintness ; before the expiration of half an hour, they rose to the former standard ; this depression and subsequent rally were repeated with each dose. The fœtal heart was distinctly heard after the labour had lasted twenty-four hours, and more feebly an hour previous to the exhibition of the ergot. The uterine contractions were increased considerably in frequency, and slightly in form by the ergot, but no effort was produced upon the progress of the child ; and in consultation with Dr. Darley and Dr. Maunsell, it was determined to proceed to deliver, as the woman was evidently losing ground. Her pulse had become very quick and slightly irregular, and fever was setting in. The catheter had been passed several times during the last thirty-eight hours, but the bladder contained scarcely any urine, notwithstanding the quantity of fluid taken. I introduced the forceps antero-posteriorly in the usual manner, and with some effort delivered the patient of a dead child, without accident. On examining the state of the after-birth, the cord separated, and on the occurrence of some draining, it became necessary to remove the placenta. The pulse at the termination were 130, and the general condition of the patient was far from favourable. However, she seemed gradually recovering for three days, when she was attacked with a violent inflammation of the womb, and an unhealthy superficial sloughing of the vagina and external parts, accompanied with fever of a typhoid type. Pulse 130, and weak. Tongue dry and covered with brown fur. Sordes about the teeth and lips. Skin hot, great thirst ; distressed countenance; pain in the belly. The uterus could be felt enlarged and hard, and pressure on the abdomen gave no pain until I felt my finger touching the uterus. Leeches were applied to the abdomen, and stupes. Vaginal injections of tepid water every two hours. Solution of acetate of lead to the vulva. Calomel and opium in large doses. Catheterism was performed three times a day. She continued in this most unfavourable state with but little change for about ten days. A blister was applied to the abdomen. The calomel was omitted as it produced diarrhœa, though fortunately the mouth became affected.

The other remedies were continued sedulously. Towards the end of this time a quantity of fetid purulent matter was discharged from the uterus, which diminished in size and became less tender. The slough of the vagina assumed a more healthy appearance, and the fever decreased until about a fortnight after the operation, when I found her one morning in a state approaching to collapse. Skin cold and clammy; pulse 100 and very feeble; extremities cold. Wine and sulphate of quinine were now freely given, with warm applications to the skin, and after a little time she rallied, and has since gradually recovered. The abdomen is insensible to pressure! a healthy white discharge continued for some time from the uterus; the sloughing of vagina and vulva healed; her pulse fell; her appetite returned, and she is now convalescent though very feeble. I fear the abstract I have given of this case is almost too short to convey an accurate impression of the violence of the attack, and the struggle of the constitution to resist it. I never saw so bad a case continue so long almost hopeless, and yet recover. There are several points of great interest which I would merely point out. I do not know whether it has been frequently noticed by others, at least it has never occurred in the practice of the Wellesley Dispensary, that we have seen a patient so long in labour, drink freely, and yet the secretion of urine so completely suppressed. The catheter was passed three times in thirty-six hours, and not more than two ounces of urine drawn off. The effect of the ergot was very striking; the immediate consequence was a sudden depression of the heart's action, followed by excitement. I have known it produce excitement before even to delirium, but I had not perceived the previous sinking of the pulse. I hope soon to be able to lay before the profession a more extended series of observations on this important subject.

I may observe 'en passant,' that Dr. Collins, the late distinguished Master of the Lying-in Hospital, mentioned to me in conversation, that he has noticed a similar depression in all cases in which he has given it. Are we to attribute the death of the child to the increased pressure exercised upon it by the uterus when stimulated into more frequent action by the ergot of rye? I have before noticed, in a report of the diseases treated at the Wellesley Institution for Females, the important assistance to our diagnosis between puerperal hysteritis and puerperal peritonitis, afforded by the tenderness on pressure. In the former it is caused *only when the fingers are felt to come in contact with the enlarged and hardened uterus;* the abdomen generally being free from pain. In the latter the tenderness is diffused and nearly equal in every part of the abdomen. As to the treatment, without diminishing the value of ordinary antiphlogistic measures, I would notice specially the advantage of repeated vaginal injections of warm water, and the exhibition of opium, either alone or in combination with calomel. It is well if the mouth can be affected, but if not, or if diarrhœa supervene, I should rely with much confidence on large and repeated doses of opium as recommended by Drs. Graves and Stokes. During the attack, I am sure the patient took nearly two scruples of solid opium without any ill effect, and with most decided benefit."

The second case, is very brief, and was published in Dr. Ryan's Journal for January, 1834.

"On the 6th of February last, I was called upon by my friend Dr. Houghton, to visit a poor woman, Mrs. Cooney,

Bass-place. She had been delivered two days, after a natural labour, and had taken cold. She complained of pain in the lower part of the abdomen. The enlarged and hard fundus of the uterus could be felt higher than natural, and pressure on this gave acute pain. The abdomen was unaffected by pressure. Pulse 120 and very weak; tongue brown and dry; sordes about the teeth; great anxiety and weakness; skin rather hotter than natural; bowels free; lochia suppressed. I ordered her a blister to the abdomen, with stupes, and gave five grains of calomel, and one of opium, every two hours. She continued in the same state several days. The mouth was not affected: the calomel was omitted, and opium given alone, in one-grain doses. She then gradually became better; a slight discharge of puriform matter took place, succeeded by the lochia. The uterus diminished in size and became less painful; the pulse fell, and the tongue appeared more natural; she gradually recovered, and is now well. Although this cannot be compared for severity with the former case, still the symptoms of uterine inflammation were very decided, as was also the relief afforded by the opium."

Since the above cases were written, another case of metritis occurred to our author, in which the uterine tenderness, and the benefit derived from opium, were equally well marked.

---

### Practical Observations on Cholera. By Mr. Henry George.

Although we entertain very few fears of cholera returning in any formidable degree to this country, yet we are bound to notice, in a concise and cursory manner, any statements that may issue from respectable sources, and bear upon the treatment of that mysterious epidemic. We may, in limine, remark, that Mr. George (a respectable medical practitioner in Kensington) considers the malignant cholera with which we have been lately visited, as a disease "closely resembling that which prevailed two centuries ago in our own island," as described by Sydenham, Etmuller, and others. In respect to the treatment of cholera, the gastric irritability is one of the most formidable symptoms.

"There is no medicine with which I am acquainted, so capable of controlling this irritability of the stomach, as the liquor potassæ given in considerable doses; I have generally administered it in the following form.

℞ Liq. potass. ʒj.; Conf. opiat. ʒss. Tinct. card. c. ʒij.; Aq. puræ ℥jss. suf. Mist. cap. dim. stat. et post horam unam repet.

And where the irritability of the stomach has been so excessive as not to bear even that quantity of liquid, I have usually given a tea-spoonful of the mixture every four or five minutes, until the urgent symptoms were abated; nor has such an effect ever failed to follow its diligent use, for it was reflection on the only fatal case of cholera which occurred in my own practice* that induced me to experiment with the medicine; but I do assert that I feel more anxious to support the principle of treatment than to insist on the exclusive employment of the remedy, useful as I have found it; for where I could not obtain the liq. potass. I have administered, even in very distressing cases, a table-spoonful of burnt brandy every few minutes with the happiest effect."

On the reaction we need not dwell. Our author entertains some peculiar opinions on this point. He does not consider this reaction in the light of

---

* "There was (independent of the case alluded to) an infant whom I attended and rescued by its use, from a state which appeared to threaten its immediate dissolution. A physician was consulted, and the opinion given, (in which I cordially agreed,) was, 'that there was nothing but debility to contend with anticipating recovery;' but the cretaceous powder with opium, kino, &c., was ordered, and the wine was discontinued; *vomiting* followed, and in a few hours the child was a corpse."

fever, but as a constitutional tumult or disturbance to be treated by stimulants, opiates, and tonics. We cannot say that our own observations have led us to the same conclusion.

---

### Renal Disease.

Some cases have lately occurred in our own practice, which have led us to reflect a good deal on the insidious approach of renal affections, their proneness to imitate complaints in other parts than the kidneys, especially in the bladder and urethra—and lastly, the slow, but fatal character of the disease.

One of the cases that particularly attracted our attention, was a gentleman turned of 50 years of age, who had, for some years past, complained occasionally of pain and uneasiness in the region of the liver, with the usual symptoms of what are termed biliary obstruction, as yellow tinge of sclerotica, high-coloured and lateritious urine, pale fæces, and general irritability, white tongue, &c. These symptoms always gave way, without difficulty, to the usual means. In the month of August, 1834, while we were on the Continent, this gentleman was seized with a new train of symptoms—the chief of which were, frequent inclination to make water, with dull aching pain extending from the neck of the bladder along the urethra, to its extremity, where the pain resembled that produced by calculus in the bladder. To these symptoms were soon added a muco-purulent deposit in the urine. The state of the general health varied much, but he never distinctly referred to the region of the kidneys as the seat of any pain. He was frequently examined with this view, but without our being able to trace the disease to any higher source than the bladder. Sir Benjamin Brodie saw the patient in December, and, as the symptoms resembled those of calculus in the bladder, the patient was sounded, but no stone found. The urine was analyzed, and a very slight trace of albumen was discovered, when boiled in a spoon. Under general treatment and regulated diet, the health greatly improved, but the local symptoms never gave way, nor the muco-purulent deposit in the urine. He got so much better, however, that he ate his meals with good appetite, took his few glasses of wine, and said he was as well as at any period of his life, were it not for the pain at and after making water. This pain lasted from half an hour to three or four hours after each evacuation. In this state he was suddenly seized with violent pain in the region of the liver, extending over the front of the abdomen, with fever, vomiting, and, in short, all the symptoms of peritoneal inflammation. The most active depletion, general and local, was employed; but enteritis was clearly established, and, on the fourth day he died. The following were the appearances on dissection, as verified by Sir B. Brodie, Mr. Bagster, and Mr. H. J. Johnson, one of the editors.

*Dissection.* On opening the abdomen there was considerable effusion into its cavity, of a liquid resembling a mixture of serum and bile. The convolutions of the intestines were also glued together by recent lymph. There appeared to be a minute ulcerated aperture in the gall-bladder, which, allowing the escape of bile, had given rise to the peritoneal inflammation. There were biliary calculi in the gall-bladder.

The urinary apparatus was carefully examined. The bladder and urethra were quite sound.

The right kidney was more vascular than natural, soft, its capsule very readily separable from its surface, and the mucous membrane of the infundibula and pelvis of the ureter vascular also.

The left kidney was still more decidedly affected, for, in addition to the marks of chronic inflammation observed in the right, the pelvis and some of the infundibula were much dilated, a calculus was situated in one of the latter, and an encysted cavity, containing puriform fluid, was found in the substance of the organ.

We do not mean to say that this case is very singular, much less that it is unique; but we believe that it is not very common to find *all the symptoms* where there was no disease—

*all the disease* where there were no symptoms. In a discussion at the Westminster Medical Society on this point, it was urged that such cases were by no means uncommon, and Mr. Howship's work was referred to as proof of this position. The two following cases were urged as being perfectly conclusive on this point.

Case 1. " *Ulceration, with Calculi in the Kidneys.**

Catharine Harwood was admitted into the Westminster Hospital, Aug. 7, 1765. She came in reported with stone, and was under Mr. Pyle's care. There was a suppression of urine occasioned, as was supposed, by a stone or stones in the bladder; but on examination none were found; while in the hospital she in general passed but little water, yet on introducing the catheter upon one occasion as much as half a pint was found. She remained much in the same state for a fortnight, and died on the 21st of the month.

*Examination.* In the abdomen all the viscera appeared sound except the kidneys, and here was the source of *all her complaints,* as well as the cause of her death.

Both the kidneys were in a soft, and almost putrid state. They were very much enlarged. In the pelvis of the kidney upon the right side, was a triangular knotty stone, one angle of which had passed through a small ulcerated hole in the pelvis, and appeared externally. The pelvis of the kidney was exceedingly thin and tender round the part where it had given way.

Lodged in the infundibuli of each kidney calculi were found; in the cells of the right were several small stones; in those of the left there was a great deal of sabulous matter, but only one stone that had reached the size of a pea. The ureter of the left kidney had several small calculi in it, for some distance down. These were not larger than small pepper corns.

In the bladder was a small quantity of sabulous matter, adhering loosely to its internal coat. Otherwise the bladder was healthy, being neither inflamed nor contracted.

The opening made by the stone through the pelvis of the kidney, must have allowed the urine to escape into the cavity of the abdomen, and thus have hastened the fatal termination of the disease."*

* " Extracted from a MS. of the late Mr. Watson's; which together with the disease, is preserved in Mr. Heaviside's museum."

Without wishing to question the truth of the foregoing statement, it will not be denied that the case is exceedingly imperfect as to the history—of which, in fact, there is no detail whatever. It is merely an *extract* from a manuscript of Mr. Heaviside's, and there is much reason to believe that if all the symptoms of the case had been narrated, some of them would have had reference to the region of the kidneys before the death of the patient. We shall, however, let that pass.

*Case* 2. The next case related by Mr. Howship is that of a boy, five years of age, whose symptoms were those of stone in the bladder, and for which he was sounded, when a stone was found. In this case, "the fits of pain and distress, *which usually commenced in or about the loins,* passing *downwards towards the bladder,* still continued to return as frequently and severely as ever, reducing him both in flesh and strength, till at length a fresh attack of excruciating pain and irritation supervened, and excited fever. He now complained of great pain and excessive tenderness over the whole abdomen. He soon sunk."

On dissection, a quantity of purulent matter mixed with urine was found in the abdomen, and which had escaped from diseased kidney. The bladder was much thickened in its coats, and contained a calculus.

In this case it does not appear that disease of the kidney was suspected; but it cannot be maintained that no symptoms indicative of such disease presented themselves during life. At all events, as there was disease in both parts of the urinary apparatus, it is impossible to say which had the pri-

* Howship, p. 40.

ority, or which excited the sympathy of its neighbour. The following case, however, is more to the purpose.

*Case* 3. "In 1794, I was sent for to see a young lady, Mrs. P——e, who had been married about a year. She became subject about five months previous to my seeing her to an irritation at the neck of the bladder. She had a very frequent desire to pass her water, night and day, the urine depositing a great quantity of thick mucus. These complaints she imputed to having taken cold during menstruation, which suddenly ceased, and never returned.

The disorder continued for six weeks, in spite of opiates, and other rational means. At this time however it suddenly left her, upon the coming on of a pain in the back, with which she was suddenly attacked. This pain was constant, and was situated in the region of the right kidney. A few days subsequent to the commencement of the pain, a tumor appeared upon the part, and continued gradually to increase, extending forwards towards the region of the liver. This gradual increase of the tumor externally went on for about two months.

In this stage of its progress, I was called upon, and found a large tumor in the region of the liver, very hard, very extensive, and in some parts evidently containing a fluid.

I said this seemed to have been *one of those cases I had sometimes seen, wherein the disease had never existed in the part where the first symptoms had appeared.* That I conceived she never had any disease in the bladder, but a symptomatic action from an original affection in the right kidney, which perhaps might have suppurated, and during the inflammatory stage, it had probably formed an adhesion to the liver, so as to point through that viscus."

We think it very probable that the original symptoms in the bladder were symptomatic of disorder in the kidney: yet we have no *certainty* that such was the case. Irritation may have been *transferred* from the bladder to the kidney, as we know it often is, from one part of the urinary apparatus to another. From the passage which we have quoted in Italics, it seems that Mr. Heaviside had sometimes seen cases where "disease had never existed in the part where the first symptoms had appeared;" though it is not quite evident that this expression applies exclusively to the urinary organs, but to other parts also.

Such occurrences as those detailed in the case noticed by ourselves, are certainly not very numerous, and are rather the exceptions than the general rules. Sir B. Brodie has not been able to verify more than two or three such instances, as appears from his valuable work.

The second case which we have to state was that of a lady about 33 years of age, who had been affected for about two years, with dull pain in the perineum, anus, and region of the sacrum, accompanied by an irritable state of the bladder. The urine did not present, for a long time, any thing extraordinary. About six months before her death a muco-purulent deposit appeared in the urine, and then the pain in making water became very severe, lasting for a quarter or half an hour after each evacuation, and appearing to be in the neck of the bladder and along the meatus urinarius, to the very extremity of the passage. These symptoms very gradually augmented in severity, and her general health began to decline. She wasted in flesh, got progressively weaker, and a very slight exertion brought the pulse up to 120 in the minute. She was carefully examined by Sir B. Brodie, Sir C. M. Clarke, Dr. Robert Lee, Dr. Prout, Dr. Johnson, and others; but no indication of any disease of kidney could be traced beyond the symptoms above-mentioned, and which referred to the bladder and urethra. The regions of the kidneys were repeatedly and carefully explored; but no pain, tenderness, fulness, or other phenomenon indicative of disease could be detected. The urine betrayed slight evidence of albumen, besides the muco-purulent deposit already mentioned. The uterus was perfectly sound, but the coats of the meatus felt somewhat thickened, and the neck of the bladder, on the right side, was tender when pressed upon by the point of the finger. No medicine appeared to have any decided effect on the complaint, and the unfortunate patient declined progressively in health and strength, having lost all appetite, and being affected with a kind of hectic fever at night. A few weeks before her death, all doubt was removed, in respect to disease being in the kidney or neighbourhood. The region of the right kidney and the hollow of the right ilium became the seat of exquisite pain and tenderness, as well as some fulness—the muco-purulent discharge changed into extremely fetid pus, and became greatly increased in quantity, the symptoms of pain in making water, and frequency of micturition continuing the same as before the apparent affection of the kidney and iliac region was developed. From the pores of the skin a clammy perspiration exuded, which had a heavy and disagreeable odour. Hectic fever became established, and the patient died. On dissection, the right kidney was found to be extensively diseased—a large abscess in the right iliac fossa—the coats of the bladder thickened, and the mucous lining ulcerated. There was no calculus.

---

### Dentology.

1. The Anatomy, Physiology, and Diseases of the Teeth. By Thomas Bell, F.R.S. Second Ed.
2. Dentologia: a Poem on the Diseases of the Teeth, &c. By Solyman Brown, A.M, New York, 1833.

These two works are each classical in its way. The first on the list has been stamped as such by public approbation—being a practical work on the anatomy and diseases of the teeth, grounded on the present advanced state of our knowledge—and that from the pen and from the experience of one of our most talented lecturers on dental pathology. It is curious that Mr. Bell has prefixed no preface to this second edition, and assigned no date to that of the first. We are, therefore, left in the dark, as to the additions or alterations which the work has undergone. We conclude, however, that no additions, with the exception of one or two notes, have been made to the present edition. In the 11th vol. of this Journal, Nos, 21 and 22 (July and October, 1833), we gave a full analysis of Mr. Bell's work, and spoke of it as it deserved. On the present occasion, therefore, we can only announce a second edition, without additions. We shall, however, extract an interesting case, introduced (we believe for the first time into this edition) in a foot-note from his friend Mr. Morgan, the distinguished surgeon of Guy's Hospital. It is given in illustration of local neuralgia, as distinguished from constitutional neuralgia, and is both curious and interesting from the circumstance, that the exciting cause of the disease was temporarily removable at the will of the patient.

"The subject of the disease, by trade a tailor, of apparently sound constitution, and between fifty and sixty years of age, was placed under my care as a patient in Guy's Hospital, in the month of May last.—He was at that time suffering from the effects of entropion in both eyes. In the left eye, the disease was producing but trivial inconvenience, whilst in that on the right side, the suffering was extremely severe, and of an unusual character. The account which he gave of the commencement and progress of his complaint was as follows:—

About six years ago, after a severe attack of ophthalmia, which was accompanied by very considerable swelling of the lids, the tarsi became inverted. In consequence of this, the distressing symptoms usually met with in cases of entropion supervened. For the relief of these symptoms he placed himself at different periods under medical treatment, but without receiving any permanent benefit from the remedies which were made use of. The formation of a slough by the application of caustic to the under part of the lower lid, and the subsequent excision of a portion of the orbicularis palpebræ, were operations to which he submitted without experiencing any beneficial result.

Until within the last two years, the symptoms which he describes, are simply those of severe entropion; but about this period a peculiar neuralgic affection was added to his other sufferings, which constitutes the principal point of interest in the case; it consisted, to use his own expression, in a 'ticking, flickering, darting pain,' which occurred occasionally during the day, and was constant when in the recumbent posture. This pain was altogether different from any which he had ever experienced before, and extended from the lower lid and globe of the eye on the right side, over the forehead, right temple, along the lower jaw, down the side of the neck and arm, to the right elbow, and occasionally also as far as the wrist.

These occasional neuralgic affections were existing in the highest degree of severity at the time he placed himself under my care; and, at this period, the cornea of the right eye was rendered partially opake and highly vascular, by the constant pressure of the inverted lid; there was severe conjunctival inflammation, and the intolerance of light was excessive.

The inversion of both lids of the right eye was considerable, the lower lid being somewhat more inverted than the upper; and the connexion between the entropion of this part and the singular nervous affection which I have mentioned, was clearly proved by the circumstance, that even in the most severe paroxysm of pain, a separation of the lower lid from the globe, produced at all times a temporary and instant relief.

It appeared to me, therefore, that the removal of the cause of his sufferings might be effected by the excision of that portion of the cartilage of the lower lid, which, by its pressure, was keeping up, if not producing, morbid excitement in the nervous system. I therefore removed about two-thirds of the inferior tarsal cartilage, by cutting out a triangular central portion of the lid. The result of this operation, however, disappointed my expectations; for, although temporary relief was afforded, yet the remaining portions of the tarsus were found, after the healing process had been completed, to produce the former train of symptoms, in consequence of their contact with the globe. I then removed, by excision, the whole of the inferior tarsal cartilage, and produced for a time a complete alleviation of his neuralgic complaint. In the course of about six weeks, however, the disease returned with its former severity, and was now referred to the inverted condition of the upper lid; for the paroxysms were invariably and instantly stopped by a separation of the superior tarsus from the eye-ball. The tarsal cartilage of the upper lid was, therefore, dissected completely out; and the operation, which was performed about six months ago, has hitherto been attended with complete success as regards the removal of the neuralgic affection. I should also mention, that soon after his first admission into the Hospital, the extension of pain along the lower jaw during the paroxysms, was entirely prevented from recurring by the extraction of three carious molar teeth on the same side.—At present, the man is suffering from chronic ophthalmia in the right eye, and from entropion in the left. Previous to the excision of the tarsi, the constitutional remedies which are occasionally found beneficial in cases of tic douloureux were tried without avail.

Yours, ever most sincerely,
JOHN MORGAN."

The second work on our list is partly from the pen of a transatlantic poet, and partly from that of an ingenious dentist (Mr. Parmly), who resided and practised for some years in this metropolis. Although the extensive subject of HEALTH generally, afforded ample scope to the muse of an Armstrong, we had little idea that DENTOLOGY would have furnished materials for an epic poem, in five cantos, by a reverend divine of the New World. Yet such is the fact; and if we cannot conscientiously place Mr. Brown on a par with Armstrong and Darwin, yet we should not be fair in withholding from him the meed of praise, for some power in versifying the unruly tortures of toothache, and the technical terms of filling, filing, and extracting decayed teeth.

" Whene'er, along the ivory disks, are seen,
The filthy footsteps of the dark gangrene;
When caries comes, with stealthy pace to throw
Corrosive ink spots on those banks of snow—

Brook no delay, ye trembling, suffering fair,
But fly for refuge to the dentist's care.
His practised hand, obedient to his will,
Employs the slender file with nicest skill;
Just sweeps the germin of disease away,
And stops the fearful progress of decay."

We have been fortunate in never having experienced personally the torture of TOOTH-ACHE; but we have heard it described by sensitive sufferers, as something beyond the power of language to delineate in its true colours. To those who have felt this pang the following lines will recall recollections of past sufferings—a source, as Virgil tells us, of great comfort, when the danger is over.

Forsan et hæc olim meminisse juvabit.

"They say who most have felt, and best should know
The power of this most execrable wo,
That when Pandora's box of mortal pains,
Was first unlock'd among the wondering swains,
To ev'ry vice its kindred grief was sent,
And every crime received its punishment,
Except intemperance:—no *single* ill
Could Heav'n's irrevocable law fulfil,
The fixed resolve, th'omnipotent decree,
That each offence should meet its penalty;
Then all these mortal woes in one were joined,
And toothache came, the terror of mankind!
Thou haggard fiend! of hellish imps the worst,
To mercy deaf, by sorrowing man accurst;
Though cheerless days made desolate by thee,
And long, long nights of sleepless agony,
Have marked thy fearful reign in days of yore,
Thy power is crushed,—thy scorpion-sting no more
Affrights the helpless, for the dental art
Commands thy gloomy terrors to depart,
Then wipes from beauty's cheek the tears that burn,
And bids her roses and her smiles return."*

The notes to the poem are by Mr. Parmly, who, as Bolingbroke furnished the materials to POPE, for his "Essay on Man," enabled the Rev. Solyman Brown to compose his Essay on the Teeth. These notes by some dull prosaic mortals, will be considered more valuable than the effusions of the Muse—we pity all such!

---

DISEASES OF THE RECTUM. MR. LISTON.

The intellectual city has lost one of its most talented surgeons and dexterous operators. We hope the London University Hospital, and the British metropolis will afford ample scope for Mr. Liston's abilities, and that the profession will benefit by the zeal which appears to be diffused among the teachers in that infant institution. No stimulus is equal to that of NECESSITY! The NEW HOSPITAL and the NEW SCHOOL cannot hope to maintain their ground, much less ripen into maturity and reputation, but by the most vigorous efforts of every individual connected with them. This conviction is evidently felt by them all—and the public will profit, not merely by the exertions of that party, but by the impulse which it will lend to every hospital and school in the metropolis. Hence the advantage of free institutions and fair competition. The state of the world, and especially of the medical profession, is such, that no individual

---

* This passage reminds us of Burns's address to the tooth-ache.

"My curse upon your venomed stang,
That shoots my tortured gums alang,
And through my lugs gi's mony a twang,
Wi' gnawing vengeance!
Tearing my nerves wi' bitter pang,
Like racking engines!

When fevers burn, or ague freezes,
Rheumatics gnaw, or cholic squeezes,
Our neighbors' sympathy may ease us
Wi' pitying moan;
But thou—the hell o' a' diseases,
Ay mocks our groan!

Where'er the place be priests ca' hell,
Whence a' the tones o' misery yell,
And ranked plagues their numbers tell,
In dreadfu' raw;
Thou—tooth-ache, surely bear'st the bell,
Amangst them a."

can hope to maintain his ground, much less to distinguish himself, without exertions almost superhuman—and it is the same with aggregations of individuals—with public bodies.

---

In a clinical lecture delivered at the University Hospital by Mr. Liston, the lecturer draws the attention of his pupils to narrowing of the anal passage, a complaint incident to the extremities of all mucous canals. "There is occasionally seen a permanent and organic stricture here, the orifice forming a hard, narrow, and unyielding ring. This arises from long-continued irritation of the part, immediate or remote, and consequent change of structure, from the cicatrization of rhagades (deep fissures); or it is seen to follow loss of substance, as after some of those unwarrantable incisions, excisions, or cauterizations, which are occasionally practised on these parts." This is not likely to be alleviated, he thinks, by further loss of substance, as has been recommended. But the contraction, in this place, is far more frequently of a spasmodic, than of an organic or permanent character, arising from irritation, either in the part itself, or in some other part with which the extremity of the gut sympathsies. One of the most troublesome cases he ever met with, arose from the lodgement of a piece of bougie in one of the sinuous tracks of the rectum. Various foreign bodies, as pins, pieces of bone, or other indigestible things that are swallowed, may pass through the whole canal, and become arrested near the anus, keeping up constant irritation there. But the most common cause is the presence of chaps or fissures in the orifice—a complaint described by Paré and Wiseman, but more recently brought to notice by Baron Boyer.

"These rhagades or clefts are met with of various depths, singly or not; the one end of them is visible, but their full extent is not ascertained, unless when the patient presses down; they generally rise in the direction of the bowel, but sometimes deep ulcerated cavities are found within the sphincter, and running transversely. Such breaches of surface no doubt frequently have their origin in laceration of the membrane, by the passage of hardened excrements or of foreign bodies. Large pieces of bone appear to pass along the whole track of the intestinal canal without difficulty or inconvenience, and are stopped by the sphincter. I have removed many (the last was nearly one half the jaw of a rabbit) from within its grasp. You can readily understand how the existence of such breaches of surface should occasion and keep up uneasiness and spasm in the outlet of the canal. The regulation of the bowels, the clearing out of the lower part of them, careful ablution, must all be attended to in such cases. The irritability of the sores may be done away with and their healing promoted by the occasional use of the nitrate of silver, in substance or in strong solution, applied by means of a camel-hair brush. The sores within can be readily seen and reached in most cases, so that remedies suited to their state may be employed."*

In some bad and intractable cases, it becomes necessary to divide the sphincter, which is readily done by a blunt-pointed straight, and narrow bistoury, conducted on the finger. The incision may be made through the fissure or ulcer; but it is not material whether the chap is involved or not in the operation. Some of the "Rectum Doctors," Mr. L. remarks, have recommended the excision of a piece of the verge of the anus, as a remedy for contraction—and this is to be done "with such a cutting scoop as cheese is cut with." We de not recollect, at this moment, the "Rectum Doctor," to whom Mr. L. directs the above sarcastic passage; but we should be sorry to employ him ourselves in any such operation. The prescription has not the advantage of homeopathic principles—that of employing *small doses* of the drug, whose effects resemble the original disease. Here, large doses of additional contraction are prescribed for that which already exists!

---

* Lancet, No. 508.

Stricture of the rectum, properly so called, is rather a rare disease. And the lecturer can afford no stronger proof of this rarity than the fact " that in all the pathological museums taken together, in this vast metropolis, you will find but very few specimens," unconnected with some specific disease, as syphilis, cancer, &c. The same holds good with respect to collections in other countries. We fear that Mr. Liston is not far wrong when he apprehends that " the frequent existence of this disease has been asserted now and then to serve no good purpose."

" It is not at watering-places only that such charlatanerie is practised; and as most invalids experience difficulties in evacuating the bowels, many become victims to the most disgusting treatment. So successful was the imposition, and so flourishing the trade, at one time, that a patient informed a friend of mine, that when he went for advice, he was ushered into a room where there were no less than eleven individuals, each with a bougie in his fundament, and with the small-clothes cut after a particular fashion, to facilitate their easy application."

Every body knows the locality to which Mr. L. alludes, and a celebrated name is seldom acquired without some *fundamental* cause. This indiscriminate practice of " Burgeeing," (as Jack Tar calls it) the rectum, is often attended with worse consequences than the mere extraction of money. The pocket of the " *malade imaginaire*" may as well be emptied by the rectum doctor, as by a colon doctor, duodenum-doctor, or even the stomach-doctor; but blue pill, and gentian draughts seldom produce such serious consequences as unnecessary poking with instruments into an irritated canal. " Not a few patients have succumbed rapidly within a very few days after the examination made with bougies and ball-probes." Sir C. Bell relates a fatal case of this kind, and Mr. Colles, of Dublin, alludes to the same subject. Our lecturer stumbled on a case where " a bougie *three feet* in length was considered necessary to reach the stricture."

" The disease to which the term of " stricture of the rectum" can with any propriety be applied, is met with immediately within the sphincter ani. It is a veritable contraction of the whole circumference of the bowel, varying in tightness, having a sharp edge, and attended with some thickening and alteration of the coats of the viscus for a little way above and below. There is a widening of the canal above, from accumulation, a profuse discharge of vitiated mucus, of muco-purulent fluid, occasionally of blood, from the contracted part, or from accompanying piles; not unfrequently there is ulceration to be discovered above the contraction. Sometimes abscess forms in the surrounding cellular tissue, and makes its way into the bowel, by the side of it, outside of the sphincter; into the vagina in the female, or some part of the urinary apparatus in the male. In some rare cases, abscess has passed through the sacro-ischiatic notch, and formed a swelling upon the hip, through the opening of which, as in the case from which this preparation was obtained, fluid fæces and flatus are discharged. The track or fistula accompanying stricture, generally is *complete*, and opens into the bowel above. The contraction is generally single, but I have met with two cases in which one ring was situated above another, a portion of sound bowel being interposed; in fact there were two distinct strictures, but both within reach of the point of the finger; the upper one could be brought within reach when the patient bore down. The diseased part can even be brought into view, though this is seldom requisite, by the use of a proper speculum, such as I hold in my hand."

The occurrence of scirrho-contracted rectum is also happily rare, for it is a dreadful disease, over which we have little control. Its pains may be mitigated by securing an easy passage daily by means of lavements. We wish success to the worthy lecturer, and plenty of patients to exercise his skill.

---

**Remarks on Endermic Medication.** By G. G. Sigmond, M.D., Physician to the Charing-Cross Hospital.

We are only able to give a concise view of this able paper, which was read before the Medico-botanical Society.

"The continental medical men have considered cutaneous absorption, or imbibition, under two heads; to the first, which consists in the simple rubbing in through the skin of medicinal substances, they have applied the term iatraleptic medicine; to the second they have given the name of endermic, and this consists in the removal of the epidermis, by such means as will not produce any structural change in the subjacent tissue, and then the introduction of a remedial agent through the denuded surface; these two systems of cutaneous medication have been carried to a very great extent."

Dr. Sigmond's description of the epidermis and true skin we must pass over.

"In a lecture delivered by Magendie, and published in the *Lancet,* we find that he performed an experiment before the whole of his class, demonstrating that as long as the epidermis is in a sound and healthy state, cutaneous absorption does not take place, unless friction be employed. He placed with the glass tube a few drops of prussic acid on the integuments of a rabbit, and the poison did not produce any appreciable effect. This is at variance with the experiments of others. M. Emmett tells us, that he found the essential oil of bitter almonds, applied to the uninjured skin of a rabbit, produce the usual symptoms, and death; and, he adds, that the peculiar odour of the poison was very perceptible in the deep-seated muscles of the back. The absorption of lead, in whatever form it comes into contact with the skin, will produce colic, or the paralysing effects which it causes when taken internally, without any apparent action upon, or destruction of, the epidermis. Arsenic when applied to the skin of the human subject, may produce all the ordinary symptoms which attend its internal administration, and, indeed, it will act with equal violence and rapidity; the stomach will exhibit the same signs of inflammation. Mercury will also cause its peculiar effects when applied to the skin. Corrosive sublimate has been known to excite, through the sound skin, an action as violent as through the alimentary canal; salivation, and even death, have been known to follow its application. Vapours can be transmitted through the skin, and Magendie lately demonstrated the fact to his class. He enclosed a rabbit in a large glass, the head remaining outside; the vapour of the concentrated acid was introduced into the glass, and the effects were manifest in about two minutes and a half; it died as quickly as if the poison had been placed on the skin or tongue. This led to an idea, that a portion may have escaped from the bottle, and been introduced into the system by the lungs.

M. Nysten found that tetanus and death were the result of leaving sulphuretted hydrogen gas for some time in contact with the skin; and Buchner on one occasion remarked, that, whilst preparing cyanogen gas, the fore-finger, which was exposed to the bubbles as they escaped, became suddenly benumbed, and that this effect was attended with a singular feeling of pressure and contraction in the joints of the thumb and elbow. We must, at any rate, all feel convinced, that substances, if in contact for any length of time with the epidermis, and more especially when aided either by animal or artificial heat, will be imbibed or absorbed. Some of them may act by mechanical destruction of the epidermis, or by some alteration in its structure. There are, besides, certain diseased states of the skin, in which absorption takes place very rapidly; thus, an infusion of tobacco applied to the head for the cure of tinea capitis has been known to produce all the bad effects which have resulted from the injudicious administration of tobacco.

Few medicines have been more fairly tried as an iatraleptic, in France, than digitalis in the cure of dropsy, and it has answered the most sanguine expectations that had been formed of its success. Dr. Chrestien, to whom we are most indebted for his experiments, has given us a fair narration of the cases which were successfully treated, and those in which it has failed. He is borne out in his practice by M. Cros Rogery, de St. Geniez, by Bernard de Beziers, by Blavet de Montbozin, by Rouchy de Montpelier, and Archbold Aspold. Under M. Rogery's treatment by the friction with digitalis, a case of dropsy of the abdomen, which followed upon a repelled eruption, was cured; under Dr. Chrestien, dropsy, the sequela of scarlatina, in a boy of five years of age, disappeared; and dropsies consequent on visceral inflammation, and on splenitis after intermittent fever, have yielded to friction upon the hypogastrium with tincture of digitalis, three times in the course of the day. The tincture is prepared by macerating for a quarter of an hour, an ounce of the leaves in three of alcohol. The method employed by Brera, which was the first introduced, and was therefore somewhat rude, consisted in macerating the digitalis in saliva, and then applying it by friction to the abdomen. In the only cases (two in number) in which I have had an opportunity of seeing it employed, I cannot say that I have seen any permanent benefit; but they have not been cases in which a favourable result could be fairly expected, as they have been desperate cases, in which all other remedies had failed. In one of these, which I have reason to believe is caused by an aneurism of the cœliac artery, and where the effusion is most rapid after the operation of paracentesis, the kidneys have lost almost all power of secretion; but, during the first two days of friction with the tincture of digitalis, prepared according to Dr. Chrestien's formula, they were evidently stimulated into some action, but soon returned to their previous torpid state.

The atropa belladonna has been tried as an iatraleptic, by Dr. Gouvion and M. Carre, in the most painful cases of neuralgia; and in rheumatism it has been found eminently successful, when its extract has been rubbed upon the abdomen, upon the sternum, or the dorsal vertebræ. In cases where, during labour, spasmodic contraction of the neck of the uterus has resisted the use of the lancet, Mad. la Chapelle says that the extract of belladonna, assisted by warm baths and emollient fomentations, rubbed upon the region of the uterus, have answered the most sanguine wishes of the accoucheur. In the reduction of hernia it has likewise been of great and important service: and also in retention of urine occasioned by muscular spasms about the neck of the bladder. M. Chevalier has published some interesting cases of spasmodic stricture cured by a bougie, on which belladonna was put. Castor oil rubbed over the abdomen produces the same effect as if taken internally, and more especially if aided by the use of the warm bath. Obstinate constipation has yielded to this remedy; and where such violent and constant sickness has been present as to preclude the possibility of the internal administration of the oil, it has produced all its good effects, without adding to the distressing state in which the stomach is found. I have seen by these means an action produced upon the bowels within a quarter of an hour after the friction had been employed, immediately on the patient leaving a bath of the temperature of 98°, where calomel, jalap, neutral salts, and lavements, had failed to relieve the intestinal canal, and where constant vomiting had commenced, and all idea of internal remedies had necessarily been abandoned. Croton oil likewise produces a complete relief of the contents of the bowels, when employed in a similar manner, and more particularly when combined with castor oil, as its general effects, when employed alone externally, are the production of a number of small red pimples, which become pustules resembling those of the small-pox, or those which are produced by tartar-emetic; and experiments have been made upon upwards of thirty patients, which prove its efficacy as a counter-irritant.

Endermic medicine consists in the removal of the epidermis by such means as cannot produce any structural change in the subjacent tissue, and then the application of a remedial agent to the surface thus denuded. Various are the modes which have been employed to expose the part on which remedies are to act; the most common practice is to apply a blister of cantharides; others prefer liquid ammonia. Boiling water has been tried, but it changes the natural structure of the skin; phosphorus has a similar effect: various other vesications have been employed, but they have been generally found to produce some irritation of the exposed surface, by which means the transmission of blood through the capillary vessels has been impeded, and thus the expected influence has been lost; the epidermis should be cut with a pair of scissors, and beneath it is to be introduced the remedial agent: if it is an insoluble substance it is to be sprinkled on the exposed surface; if it is a fluid it may be applied by moistening a small piece of lint with it. The seton is another mode by which it may be introduced, but great care and caution are requisite. Magendie gives us an instance of its bad effects in an old curé, who was poisoned in this manner by the introduction of a morsel of strychnine into a seton. The effects, in many instances, are exhibited in a very brief space of time, and the rapidity with which they are absorbed is such as to demand the greatest attention, more particularly as very minute doses will operate very readily, so much so, that very serious results have followed the application of arsenic and other remedies to denuded surfaces.

The French adopt the following plan:—Where an instantaneous blister is necessary, cut a piece of cotton, of linen, or of paper, of the size and shape for which it may be required; immerse this in spirits of wine, in strong brandy, or in eau de Cologne, lay it on the surface to be blistered, wiping the edges, so that none of the fluid may moisten the surrounding parts; apply a lighted candle rapidly over the whole surface, that it may all be burnt immediately. The ignition is exceedingly quick, and the cuticle will be found separable from the subjacent cutis.

Quinine, as an endermic remedy, has been tried with great success in the cure of intermittent fever. M. Martin published, in the *Archives Generales*, an essay on this subject. He found that thus introduced into the system, even in very small quantities, it arrested the progress of the disease. M. Lambert next tried it, and succeeded, and the practice has now become general. If a blister be applied to one of the lower extremities, and when the skin be sufficiently raised, a very small quantity of the sulphate of quinine be applied, in about ten minutes a sensation of gentle heat will be perceived in the limb, ascending to the back, and gradually diffused over the whole system. The hot fit comes on before its usual period, and the whole of the paroxysm is shortened. Even the introduction of ten grains has been found sufficient to arrest the progress of an ague. Care must be taken to reduce the quinine to a very fine powder, and to incorporate it with simple cerate, or some greasy matter, for without this a high degree of irritation will be produced. Dr. Stokes tells us, that one of the most severe and troublesome forms of ulceration he had witnessed for a long time, occurred in a case in which quinine had been applied to a blistered surface. Over the whole extent of skin to which it had been applied numerous ulcerations took place, which resisted, for the space of six weeks, every attempt to heal them. It appears that quinine is one of the most valuable of the endermic remedies, for it can be introduced where gastric irritation prevents its internal administration; it acts more quickly than through the stomach, and small quantities only are required.

Some of the narcotic medicines have been employed most beneficially in the endermic way. M. Lesieur, who published a treatise upon the 'Nouvelle Medication par la voie de la Peau,' relates seventeen cases in which he tried the acetate of morphine at the Hospital Cochin and the Bicetre; of these, four were chronic catarrhal affections which were quickly relieved, and soon cured by the application of acetate of morphine to a blistered surface; half a grain, gradually increased to two grains, was introduced; this was continued for a month, and whenever the treatment was intermitted the symptoms returned. Two cases of phthisis pulmonalis were relieved, but the dose was necessarily smaller. Pleurodynia, which resisted leeches, blisters, and other remedies, was thus relieved; a neuralgic affection of the temple was cured by the same means. A very interesting case occurred at La Pitié, in February, 1826, the details of which are to be found in the July number of the *Medico-Chirurgical Review*, condensed from the *Archives Generales de Medecine.* It was one of those gastralgic affections, which simulate gastritis of a chronic kind, attended with perpetual vomiting, and in which no internal remedy could be employed. M. Lambert applied gr. ss. of acetate of morphine to the blistered surface; in a few minutes the vomiting ceased, as if by magic, and the patient passed a better night than she had done for some time. The next day the process was repeated, and the patient slept the whole of the day; the acetate of morphine was gradually increased, until at length food was retained, and a cure effected. M. Bally, in the Memoires de l'Academie Royale de Médecine, details its effects on rheumatisms, lumbago, and sciatica, and he states that three cases of tetanus were treated by it with success; one was traumatic, the second produced by strychnine, and the third by fright.

M. Magistel has made public the happy result of his employment of acetate of morphia in hemicrania. Dr. Stokes, of Dublin has been successful in two cases of intermittent hemicrania, which soon yielded to this treatment.

M. Bally has employed strychnine in this manner with considerable success. Of two men affected with paralysis of the hands, one was evidently cured by the application of a grain to a grain and a half of strychnine daily to the raw surface produced by a blister; the second recovered the use of one hand, and nearly recovered that of the other, and a third paraplegic patient was able to walk. M. Lesieur has applied powdered strychnine, one-sixth of a grain, to the blistered surface for hemiplegia; when the quantity was increased to two grains, a paroxysm of tetanus supervened, which was dissipated by the substitution of acetate of morphine for the strychnine.

Mr. Foote tells me that he has had, at the Royal Westminster Ophthalmic Hospital, an opportunity of seeing amaurosis treated by strychnine endermically employed; a small blister was applied to the temple, and the epidermis removed, after which a quarter of a grain of the alkali was sprinkled on the sore; the dose was gradually raised to a grain: during its exhibition some of the peculiar symptoms attendant on strychnine became evident, namely, spasmodic twitchings, &c. In one or two cases it appeared to do good; vision certainly improved, but it was not pursued far enough to effect a cure; of course the danger attendant upon the employment of larger doses would naturally prevent the full use of this active substance, more particularly when the fact of the death of Majendie's curé by a morsel of strychnine endermically introduced is so well known. Very few purgatives have as yet been tried according to this method, but it has been found that tincture of aloes, applied to a denuded surface, will act upon the bowels quite as copiously as if taken internally, and many of this class of medicines act with a rapidity quite equal to that celerity with which the bowels act. It has been known that ʒij. of colocynth, or bitter apple, applied to the wound of a dog, has destroyed it with great rapidity. The necessity for the administration of endermic purgatives has not yet arisen, and therefore they have been less employed than the narcotics, from which so much benefit has been derived. Musk, belladonna, datura stramonium, hyosciamus, have been all applied in neuralgia, and with evident success, and the names of Bloquiet, Piorry, and Trousseau, are sufficient testimonies of the truth of the power of these remedies."

---

AN EXPOSITITION OF THE NATURE, TREATMENT, &c. OF FEVER. By HENRY M'CORMAC, M.D. Octavo, pp. 202. Longman and Co. 1835.

Dr. M'Cormac, who is a studious, intelligent, and observant physician, has constructed a work which bids defiance to analysis—and, strange to say, to *criticism* also. It defies analysis, because it is itself a most elaborate analysis of all that has been said, done, and even thought of fever since the creation of the world down to the æra of reform, in our days. Having thus assumed the office of analytical reviewer, Dr. M'Cormac would be offended, and justly so, if we, as brother-reviewers, took up the pen of criticism, and attempted to question the judgments, the dogmas, or the decisions of one of our own fraternity. The most ferocious animal that roams the deepest forests will not prey on its own species—and will scarcely touch the carcase when dead. We will not, therefore, set a dangerous example, lest our arms should, one day, be turned against ourselves—lest the puissant WE—the venerable Æachus, Minos, and Rhadamanthus should, some day, be bearded on their own benches, and their decisions set aside by some ruthless innovater on the wisdom of their forefathers. We are not speaking metaphorically, but literally the truth. The work is an analytical review of all that is connected with fever, its causes, nature, treatment, and prevention, guided by the light of extensive personal experience; the author having practised in "three different quarters of the globe"—and being now connected with a fever hospital in Belfast.

The prominent features of the author's, or rather the reviewer's doctrines are—that the primary link or lesion in fever is "innervation, varying in amount, and productive of all the different phenomena which distinguish the frame of one who suffers under this disease, from that of another, in the enjoyment of perfect health." In short, that the remote or invisible cause or causes of fever, act primarily on the nervous or sentient system, in consequence of which one or *all* of the functions with which the nerves have any thing to do, become deranged. They do not, in general, become all equally affected. More frequently one organ suffers in a greater degree than another, and thus have arisen the monophlogistic doctrines of Broussais and Clutterbuck. Dr. M'C. supposes also that the blood is deteriorated, and thus he presses as much as he deems proper of the humoral pathology into his service. The reviewer, in fact, is an eclectic, in the true sense of the word, and, without adopting any one system exclusively, culls from all doctrines and practices whatever appears to him both rational and useful. The thread by which these selections are woven together, is strong and silky, constituting no inconsiderable portion of the whole texture, and that, too, fresh from the wheel or engine. As a reviewer, Dr. M'Cormac deserves great praise for adopting the "fortiter in re," as well as the "suaviter in modo." There is but one other remark to be made—his general acquaintance with German writers has enabled him to cull largely from a field, but little open to English writers.*

* Dr. M'C. speaks very highly of a little German book, the "*Gesundheitkatechismus,*" or Catechism of Health, by Dr. Faust, and strongly recommends its translation into English. Is not this the work which the Quarterly Review brought forward, as the source from which Dr. Granville drew much of the materials for his Catechism of Health?

